# The Machinery at Endoplasmic Reticulum-Plasma Membrane Contact Sites Contributes to Spatial Regulation of Multiple *Legionella* Effector Proteins

**DOI:** 10.1371/journal.ppat.1004222

**Published:** 2014-07-03

**Authors:** Andree Hubber, Kohei Arasaki, Fubito Nakatsu, Camille Hardiman, David Lambright, Pietro De Camilli, Hiroki Nagai, Craig R. Roy

**Affiliations:** 1 Section of Microbial Pathogenesis, Yale University School of Medicine, New Haven, Connecticut, United States of America; 2 Research Institute for Microbial Diseases, Osaka University, 3-1 Yamadaoka, Suita, Osaka, Japan; 3 School of Life Sciences, Tokyo University of Pharmacy and Life Sciences, Horinouchi, Hachioji, Tokyo, Japan; 4 Department of Cell Biology, Yale University School of Medicine, New Haven, Connecticut, United States of America; 5 Program in Molecular Medicine and Department of Biochemistry & Molecular Pharmacology, University of Massachusetts Medical School, Worcester, Massachusetts, United States of America; Purdue University, United States of America

## Abstract

The Dot/Icm system of the intracellular pathogen *Legionella pneumophila* has the capacity to deliver over 270 effector proteins into host cells during infection. Important questions remain as to spatial and temporal mechanisms used to regulate such a large array of virulence determinants after they have been delivered into host cells. Here we investigated several *L. pneumophila* effector proteins that contain a conserved phosphatidylinositol-4-phosphate (PI4P)-binding domain first described in the effector DrrA (SidM). This PI4P binding domain was essential for the localization of effectors to the early *L. pneumophila*-containing vacuole (LCV), and DrrA-mediated recruitment of Rab1 to the LCV required PI4P-binding activity. It was found that the host cell machinery that regulates sites of contact between the plasma membrane (PM) and the endoplasmic reticulum (ER) modulates PI4P dynamics on the LCV to control localization of these effectors. Specifically, phosphatidylinositol-4-kinase IIIα (PI4KIIIα) was important for generating a PI4P signature that enabled *L. pneumophila* effectors to localize to the PM-derived vacuole, and the ER-associated phosphatase Sac1 was involved in metabolizing the PI4P on the vacuole to promote the dissociation of effectors. A defect in *L. pneumophila* replication in macrophages deficient in PI4KIIIα was observed, highlighting that a PM-derived PI4P signature is critical for biogenesis of a vacuole that supports intracellular multiplication of *L. pneumophila*. These data indicate that PI4P metabolism by enzymes controlling PM-ER contact sites regulate the association of *L. pneumophila* effectors to coordinate early stages of vacuole biogenesis.

## Author Summary

The intracellular pathogen *Legionella pneumophila* encodes at least 270 effectors that modulate trafficking of the pathogen-occupied vacuole. The mechanisms by which effectors are controlled in host cells are of key interest. Spatial and temporal regulation of effector function has been proposed to involve effector binding to host phosphoinositides. We present results showing that *L. pneumophila* utilizes the host kinase PI4KIIIα to generate PI4P on the bacterial vacuole and this signature mediates the localization of DrrA and subsequent recruitment of the GTPase Rab1. Additionally, it was found that the host PI4P phosphatase Sac1 was involved in consuming PI4P on the vacuole, which reduced DrrA-mediated recruitment of Rab1 to the LCV. Our data supports the recent concept that PI4KIIIα is important for generation of the plasma-membrane pool of PI4P in host cells, and demonstrates a functional consequence for PI4P-binding by an *L. pneumophila* effector protein.

## Introduction

Intracellular pathogens co-opt host processes to facilitate pathogen survival and replication within the eukaryotic host. To do this, many bacterial pathogens possess secretion systems that transport bacterial proteins into host cells to orchestrate their intracellular survival. After uptake by phagocytic host cells, the gram-negative bacterium *Legionella pneumophila* prevents transport of the vacuole in which it resides through the canonical endocytic pathway, and actively transforms this vacuole into an organelle that supports bacterial replication. Specifically, endoplasmic reticulum (ER)- derived vesicles are rapidly recruited to the plasma membrane (PM) -derived vacuole in which *L. pneumophila* resides after uptake by host cells [1]. These early secretory vesicles originating from the ER appear to fuse with the *L. pneumophila* vacuole [2]–[4]. Functional interactions between PM-localized t-SNAREs present on the *L. pneumophila-*containing vacuole (LCV) and ER-localized SNARE Sec22b likely contribute towards this remodeling process [5], [6].

Effector proteins delivered by the Dot/Icm secretion system contribute to trafficking and remodeling of the vacuole containing *L. pneumophila*. The protein DrrA (also known as SidM) is an effector with guanine nucleotide exchange factor (GEF) activity that directly activates the host GTPase Rab1 [7], [8]. Rab1 is an important regulator of membrane transport in the early secretory pathway of eukaryotic cells [9]. The GEF domain of DrrA (amino acids 340–533) is necessary for recruitment of Rab1 to LCVs [7], [8], [10]–[12]. Recent data indicate that activation of Rab1 on the *L. pneumophila*-containing vacuole is sufficient for promoting the tethering and fusion of ER-derived vesicles with this plasma membrane-derived organelle [13].

DrrA is found associated with the LCV membrane within the first hour of infection, but dissociates from the LCV at roughly 4 hours post-infection, which is when remodeling of the LCV to an ER-like organelle is evident [1], [10], [14], [15]. The temporal association of DrrA on the LCV membrane illustrates how the timing, localization and function of effector proteins is likely to be important for coordinating events that mediate biogenesis of the specialized vacuole in which *L. pneumophila* replicates. This is further demonstrated by the discovery of additional proteins that tightly regulate Rab1 recruitment and function during *L. pneumophila* infection. After Rab1 is recruited onto the early LCV by the GEF activity of DrrA, Rab1 function is modulated by *L. pneumophila* effectors that posttranslationally modify the switch II region of the GTPase. Rab1 is AMPylated by an adenylyltransferase domain located in a domain at the amino terminus of DrrA protein or phosphocholinated by the effector protein AnkX [16], [17]. These modifications appear to affect Rab1 function by limiting interactions with several host cell proteins that associate with Rab1 [18], [19]. A de-AMPylase activity associated with the effector SidD was found to remove the AMP moiety from Rab1 [14], [20], [21] and the protein Lem3 has a dephosphocholination activity that can remove the phosphocholine moiety from Rab1 [22]. These effectors render Rab1 susceptible to inactivation by the effector LepB, which facilitates removal of Rab1 from the mature LCV [14]. It is believed that the activities of these proteins are coordinately regulated *in vivo* by spatial cues that signify changes in vacuole morphology, but the cellular signaling mechanisms controlling these processes remain poorly understood.

Spatial localization of DrrA in host cells involves a C-terminal region of the protein that possesses phosphatidylinositol 4-phosphate (PI4P)-binding activity [8], [23]–[26]. Phosphatidylinositol phosphates (PIPs) are negatively charged phospholipids displayed on the cytosolic face of cellular membranes. Production of specific PIP species is spatially and temporally regulated by kinases and phosphatases, and the seven different PIP species are considered markers of specific cellular compartments. Importantly, these PIPs act as regulators of cellular processes due to their ability to aid in the targeting of PIP-binding proteins to discrete membrane surfaces within cells [27]–[30]. Numerous conserved domains have been shown to confer specific PIP binding properties to proteins [31]. These include pleckstrin homology (PH), FYVE (acronym of Fab1, YOTB, Vac1 and EEA1), and phox homology (PX) domains. Domains shown to bind to PI4P include PH, Tom1 (GAT), and epsin N-terminal homology (ENTH).

An increasing number of bacterial pathogens possess effectors that are able to mimic host enzymes involved in generation of specific host phosphoinositides required to facilitate bacterial survival by modulation of cellular membranes utilized by the pathogen [32], [33]. Vacuoles containing *L. pneumophila* isolated from the protozoan host *Dictyostelium discoideum* stain positive with an anti-PI4P antibody and with a GST-FAPP1 PH domain-containing probe [34], and the effector SidC that also binds to PI4P *in vitro* localizes to vacuoles during infection [35]. Thus, it is likely that PI4P is an important determinant that regulates the activities of both host and effector proteins on the LCV, including DrrA. However, the precise role PI4P plays in modulating effector localization and the origin of the PI4P residing on the LCV membrane remain unclear. The *L. pneumophila* effector protein SidF, which has lipid phosphatase activity [36], is a bacterial determinant that participates in PIP metabolism. *In vitro* assays demonstrated that SidF can hydrolyse the D3 phosphate of PI(3,4)P_2_ and PI(3,4,5)P_3_, which would facilitate PI4P production. Indeed, it was found that Δ*sidF* mutant *L. pneumophila* reside in vacuoles with reduced levels of SidC association, which supports the hypothesis that SidF has a role in maintaining PI4P levels on the LCV membrane.

Although the Golgi apparatus is the most prominent organelle staining positive for PI4P, the PM, ER and endosomes also contain PI4P [37]. Control of PI4P production is achieved by the subcellular distribution of phosphatidylinositol 4-kinases, which based on sensitivities to adenosine and wortmannin are grouped as type II and type III, respectively [38], [39]. The levels of PI4P on specific compartments is further regulated by the conversion of PI4P to PI(3,4)P_2_ or PI(4,5)P_2_ by specific kinases, and by dephosphorylation of PI(4,5)P_2_ to PI4P or PI4P to PI by phosphatases. Although progress has been made in determining the roles of PIP-metabolizing enzymes in the generation of specific pools of PI4P, most of the known PI4P-binding probes primarily localize to the Golgi-apparatus [40]–[43]. This abundant pool of PI4P is largely generated by the *cis/medial* Golgi-localized PI4KIIIβ (encoded by *PI4KB* and *Pik1* in mammals and yeast, respectively) [44]–[46]. Because the majority of PI4P probes target the Golgi-localized pool of PI4P it has been difficult to track the outcome of perturbation, by chemical inhibition or siRNA knockdown, of PI-phosphatases or kinases on less abundant pools of PI4P. Despite this challenge several groups have demonstrated that PI4P is an important determinant at the PM [38], [47], and identified the host kinase PI4KIIIα (encoded by *PI4KA* and *Stt4* in mammals and yeast, respectively) as important for generation of the PM pool of PI4P in eukaryotes [38], [40], [48]. In yeast Stt4 has been reported to localize at phosphoinositide kinase (PIK) patches that are stable cortical membrane sites enriched in Stt4 [49]. These sites have been proposed to reside at membrane contact sites linked to the ER to coordinate lipid transfer and signaling events between these two organelles [49], [50]. In support of this, after interaction with oxysterol-binding homology (Osh) proteins, which act as sensors of PI4P levels, the ER-localized Sac1 phosphatase acts in *trans* at ER-plasma membrane contact sites to reduce PM PI4P levels [51]. In mammalian cells ER-localization of PI4KIIIα was reported [46], but recent direct visualization studies have show localization of PI4KIIIα at the PM [48].

Importantly, the PI4P-binding domain of DrrA localizes to the PM when expressed ectopically [8], suggesting that *L. pneumophila* effectors may have evolved to recognize this PM-localized pool of PI4P and that this signature may be used for the localization of effectors to the LCV membrane. Thus, we set out here to investigate the *in vivo* role for this effector-encoded PI4P binding domain in an effort to understand the origin and function of PI4P during *L. pneumophila* vacuole transport, and to determine if *L. pneumophila* subverts the host machinery used to maintain PI4P levels in the cell to temporally regulate effector localization.

## Results

### Lpg1101 and Lpg2603 have a domain that constitutes a conserved *Legionella* Effector PI4P-binding Region (LEPR)

**Figure 1:**
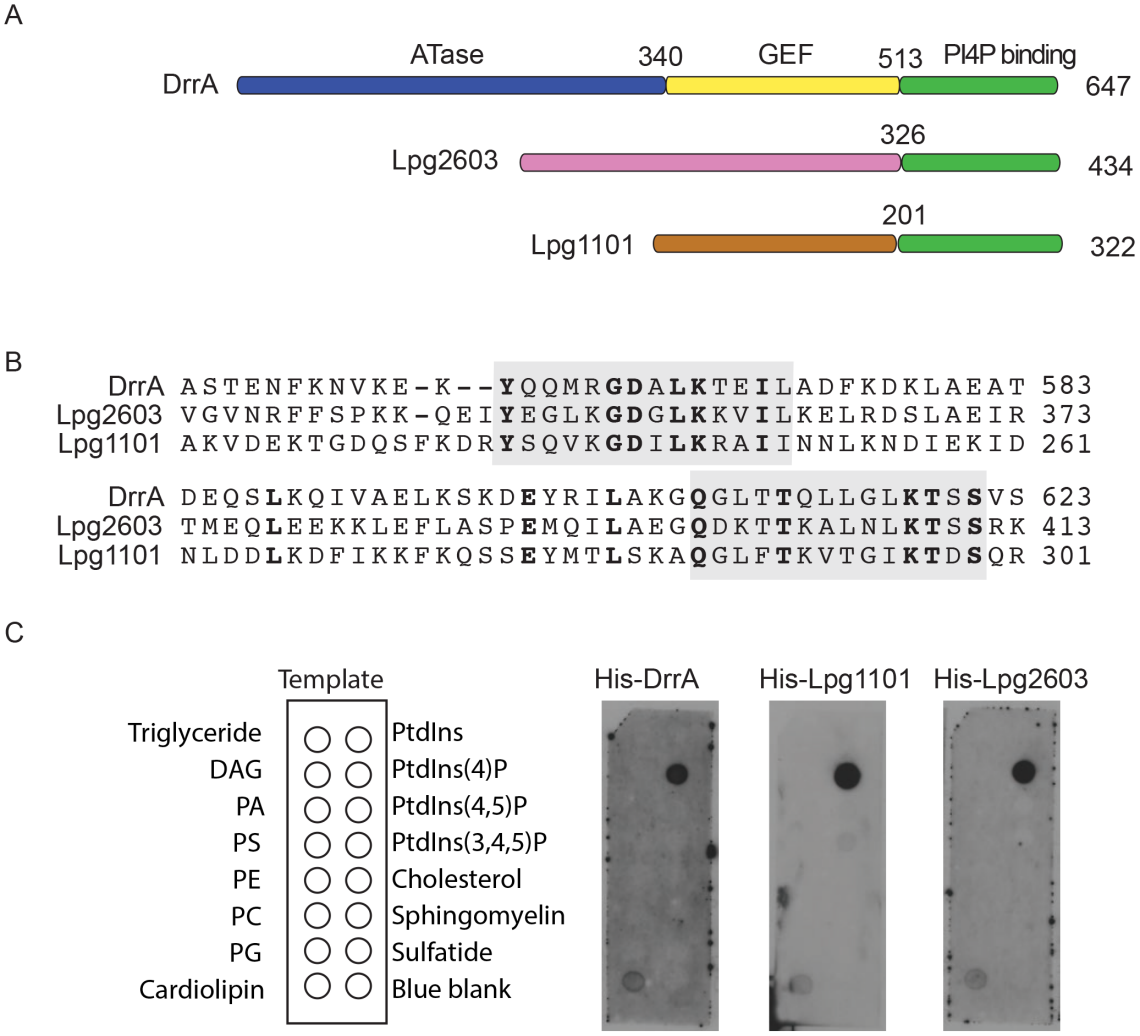
A conserved *Legionella*-effector PI4P-binding region. (A) Schematic representation of the effector proteins DrrA, Lpg1101 and Lpg2603. Indicated are regions correspond to the ATase (blue), GEF (yellow) and PI4P-binding domain (green) of DrrA. The C-terminal regions of Lpg1101 and Lpg2603 (green) have homology to the PI4P-binding region in DrrA. Non-homologous regions of unknown function in Lpg1101 (brown) and Lpg2603 (pink) are indicated. (B) Alignment of the LEPR regions in DrrA, Lpg1101 and Lpg2603 reveals conserved amino acid residues corresponding to the PI4P-binding pocket of DrrA (grey). (C) Binding of His-tagged DrrA, Lpg1101 and Lpg2603 to lipids immobilized on nitrocellulose membranes was detected using an anti-His antibody. Template shows the location of specific lipids. All three proteins showed specific binding to the spot containing PI4P. DAG, Diacylglycerol; PA, phosphatidic acid; PS, phosphatidyl serine; PE, phosphatidylethanolamine; PC, phosphatidylcholine; PG, phosphatidylglycerol; PtdIns, phosphatidylinositol; PtdIns(4,5)P_2_, phosphatidylinositol 4,5 di-phosphate; PtdIns(3,4,5)P_3_, phosphatidylinositol 3,4,5 tri-phosphate.

The *L. pneumophila* proteins Lpg1101 and Lpg2603 were predicted to have a C-terminal region that was highly similar to the C-terminal region in DrrA, and amino terminal regions with no significant amino acid homology to proteins of known function (Figure 1A). A calmodulin-dependent adenylate cyclase (Cya) assay was used to show that both Lpg1101 and Lpg2603 could be delivered into host cells by the *L. pneumophila* Dot/Icm system, suggesting these proteins are both effectors (Figure S1A). When these proteins were aligned, two regions were identified with a very high degree of amino acid identity to residues in the DrrA PI4P-binding site (Figure 1B). Unlike DrrA, the effector Lpg1101 did not bind to Rab1 *in vitro*, consistent with the homology between the two proteins being outside of any known Rab-interacting regions (Figure S1B). Consistent with the PI4P-binding activity of DrrA being contained in this region of homology, both Lpg1101 and Lpg2603 showed specific binding of PI4P using *in vitro* lipid binding assays (Figure 1C). Thus, all three effector proteins have a C-terminal region that mediates PI4P binding, which means that this domain constitutes a conserved *Legionella* Effector PI4P-binding Region (LEPR).

To assess whether the LEPR effectors affect *L. pneumophila* intracellular growth or possess redundant functions during infection, mutants lacking the individual effectors or the triple mutant Δ*1101*Δ*2603*Δ*drrA* were assessed. Mutants were not found to have an impact on intracellular replication in THP-1 cells, murine bone marrow-derived macrophages or amoeba (Figure S2).

### The LEPR confers effector localization to PM-derived structures

**Figure 2:**
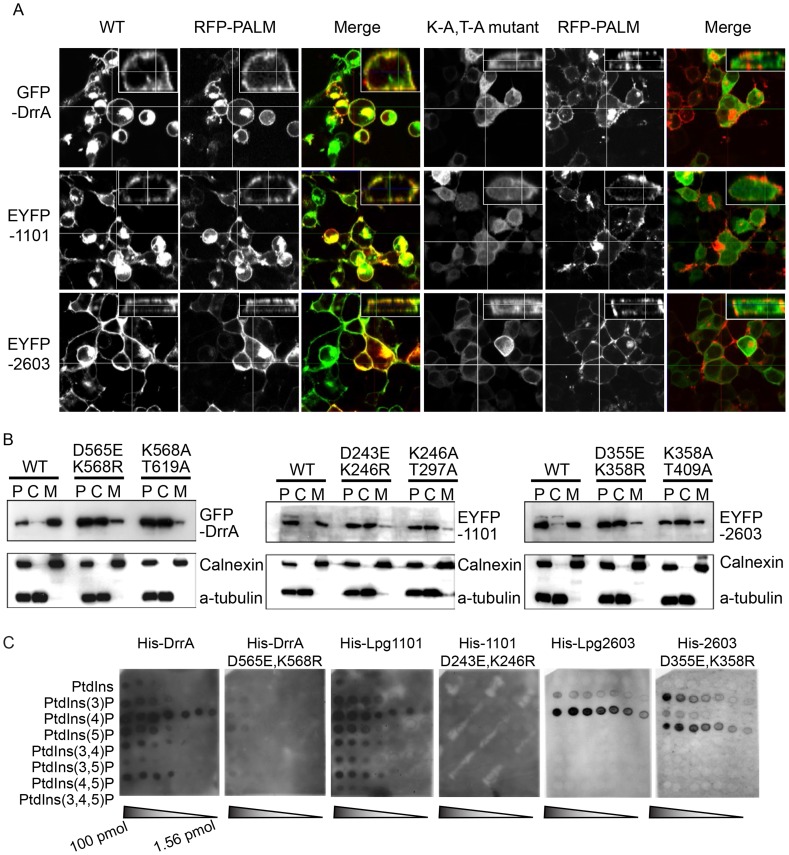
A functional LEPR region is important for localization of effectors to the host PM. (A) Orthoview micrographs representative of the localization of the indicated fluorescently tagged DrrA, Lpg1101 and Lpg2603 proteins in HEK293 cells. The large image contains a confocal xy-section and the insert panel shows a z-section through the cell indicated by the intersection of the x-axis and y-axis lines. The three left panels show the localization of the wild type (WT) protein, and the three right panels show the localization pattern observed for proteins having alanine substitutions to the conserved lysine and threonine residues in the LEPR corresponding to positions K568 and T619 in DrrA (K-A, T-A mutant). Cells were co-transfected with a plasmid producing an RFP-protein containing a palmitoylation sequence (RFP-PALM) to label the plasma membrane. (B) Fractionation of HEK293 cells expressing GFP- or EYFP-tagged effector proteins shown in (A). Note the shift in the population of effectors from the membrane fraction towards the cytosolic fraction in cells expressing the mutant versions of the effectors. A shift in the fractionation profiles is not observed in the control blots for the membrane protein calnexin or the cytosolic marker protein alpha tubulin. P = pellet, C = cytosol, M = membrane. (C) Lipid overlay experiment to determine the relative binding affinities of wild type LEPR-containing proteins versus proteins with mutations in their LEPR domains. Hydrophobic membranes spotted with a concentration gradient of eight phosphoinositides (PIP arrays) were probed with the purified His-tagged DrrA, Lpg1101, or Lpg2603 and LEPR mutant protein variants indicated. Bound protein was detected with an anti-His antibody.

Previous data indicated that the LEPR of DrrA mediates localization of the protein to the PM of host cells [8]. We confirmed that amino acids 501–647 of DrrA were sufficient for PM localization when ectopically expressed in host cells (Figure S1C). This localization pattern was somewhat unexpected considering that many proteins with PI4P-binding domains localize primarily to the Golgi apparatus. To determine whether PM localization is a general feature of effectors containing a LEPR, the localization of Lpg1101 and Lpg2603 was examined (Figure 2A panel 1, S1D and S1E). Indeed, PM localization was observed for both effectors. In addition to mediating PI4P binding, the LEPR of DrrA also mediates an interaction with PM-localized syntaxins [13], which could contribute to DrrA localization to the PM. As shown previously, the LEPR region of DrrA co-immunoprecipitated with PM syntaxins Stx2, Stx3 and Stx4 (Figure S3A), but Lpg1101 or Lpg2603 did not co-precipitate with PM syntaxins (Figure S3B). Unlike DrrA, Lpg1101 and Lpg2603 did not mediate recruitment of Rab1 to the PM, consistent with Lpg1101 and Lpg2603 having no homology to the Rab1-binding region in the GEF domain of DrrA (Figure S3C). Mutations were introduced into highly conserved amino acids in the LEPRs to determine if these residues were critical for PM localization (Figure S4). Conservative substitutions (D→E and K→R) corresponding to D565 and K568 in DrrA were made in DrrA, Lpg1101, and Lpg2603; and alanine substitutions were made to conserved residues found in the PI4P-binding pocket of DrrA corresponding to K568 and T619. These mutations perturbed membrane targeting for all three effectors (Figure 2A). Localization to the PM as determined by fluorescence microscopy was affected by these mutations, and cell fractionation studies indicated that these mutations affected the distribution of each protein from the membrane fraction to the cytosolic fraction (Figure 2B). Importantly, the D→E and K→R mutations in DrrA, Lpg1101 and Lpg2603 perturbed binding of the purified His-tagged effectors to PI4P on immobilized lipid arrays (Figure 2C). Thus, the conserved residues in the LEPR are required for PI4P binding and are important for interactions between these effector proteins and lipids on the host PM.

**Figure 3:**
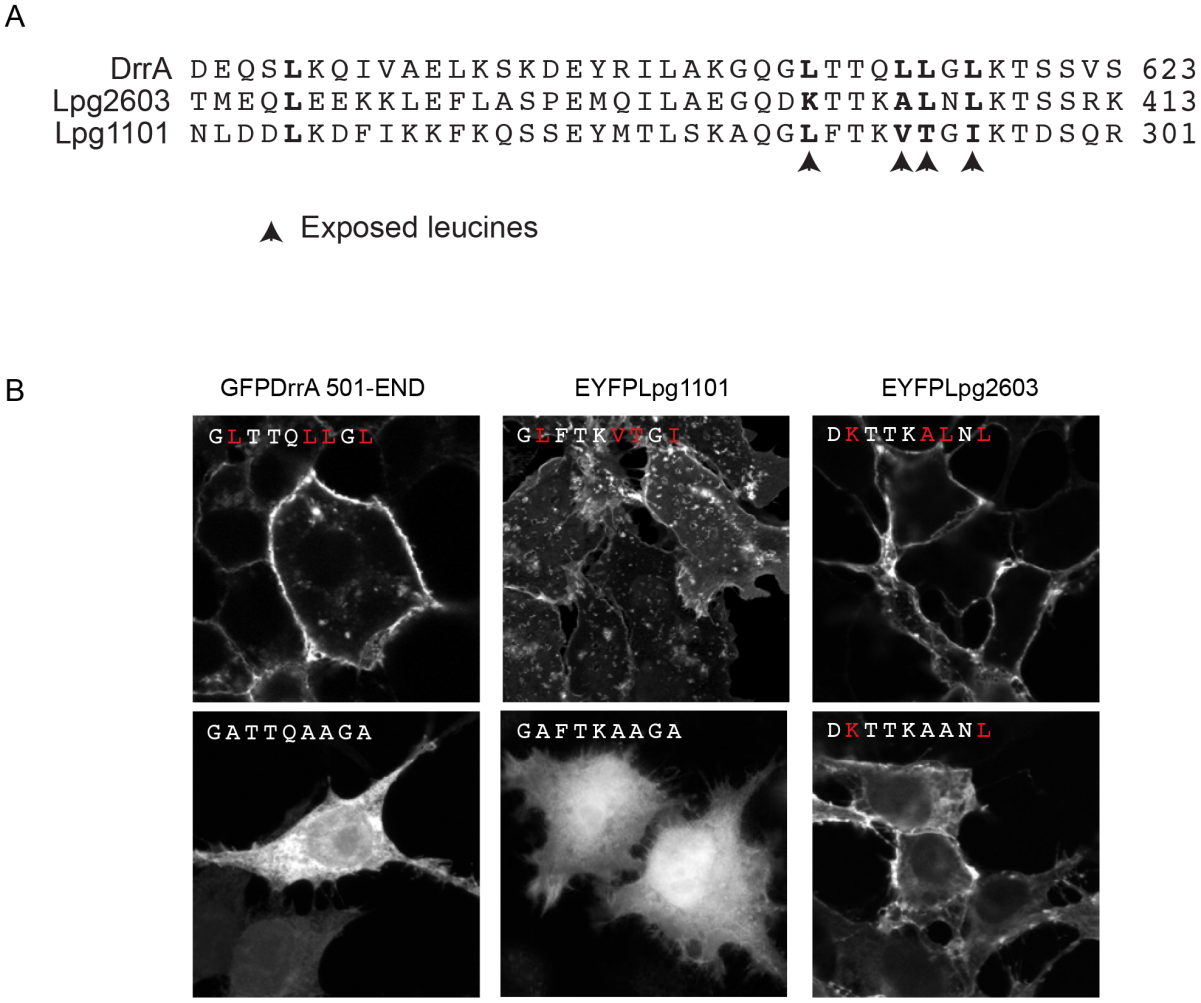
Role of the Membrane Insertion Motif (MIM) in LEPR-containing effectors association with the PM. (A) Alignment of amino acid region containing the exposed leucine residues of the MIM motif from DrrA with the similar region from the LEPR-domain containing effectors Lpg1101 and Lpg2603. The key exposed residues are indicated with arrows. (B) Z-stack micrographs of confocal images of HEK293 cells expressing GFP or EYFP-tagged fluorescent LEPR effectors and exposed leucine* to alanine mutants (*wild type exposed residues shown in red).

In addition to binding PI4P, structural studies have identified a membrane insertion motif (MIM) that is comprised of conserved polar residues lining the PI4P binding pocket in the LEPR domain [52]. *In vitro* studies have shown that upon PI4P binding the MIM region contacts acyl chains in the core of the lipid membrane, which stabilizes the interaction between the LEPR domain and liposomes displaying PI4P. To address whether membrane penetration by the MIM domain is important for PM localization *in vivo* we tested LEPR domain mutant proteins in which conserved leucine residues in the MIM domain were substituted for alanine (Figure 3, Figure S5, S6). Single and double amino acid substitutions L610A, L617A and L614A/L615A in the MIM domain of DrrA retained PM localization. The triple mutant L610A/L614A/L614A was localized primarily to the cytosol, however, some peripheral localization was still detected (Figure S5). A second DrrA triple mutant defective in three MIM domain leucine residues L614A/L615A/L617A and a quadruple MIM mutant L610A/L614A/615A/L617A displayed cytoplasmic localization (Figure S5). Likewise, an Lpg1101 quadruple MIM mutant L288A/V292A/T293A/I295A displayed cytoplasmic localization (Figure 3, Figure S6). Systematic analysis of each of lysine residues within the MIM domain of Lpg2603 was not performed because it was found that a single L405A substitution was sufficient to shift the localization of the Lpg2603 LEPR domain from the PM to the cytosol. This was in contrast to the DrrA- and Lpg1101-LEPR domains with single or double substitutions in MIM domain residues, which did not result in a profound defect in PM localization. Thus, these data indicate that membrane penetration by the MIM domain contributes to PM localization of LEPR domain-containing effectors.

### PI4P binding is important for the *in vivo* function of DrrA

Although an important role for the LEPR can be inferred from data showing this region is sufficient for PI4P binding *in vitro*, it is unknown whether PI4P binding is critical for the functioning of LEPR-containing effectors *in vivo*. Thus, assays that measure DrrA localization and function were used to address this question. The effector DrrA demonstrates strong localization to LCVs at 1 hour after infection of mammalian cells [7], [8], [10] and PI4P has been identified on LCVs isolated from *Dictyostelium* using both PI4P-specific antibodies and the PI4P-binding protein FAPP1 [34]. Consistent with data in *Dictyostelium*, we observed localization of GFP-FAPP1 to vacuoles containing wild type *L. pneumophila* in mammalian cells at 1 hour post-infection, but not vacuoles containing a Δ*dotA* mutant (Figure S7A); and GFP-FAPP1R18L, which is deficient in PI4P binding [53], was not observed in association with LCVs (Figure S7B). Thus, DrrA and PI4P are both displayed on vacuoles containing wild type *L. pneumophila* at 1 hour post-infection in mammalian cells.

**Figure 4:**
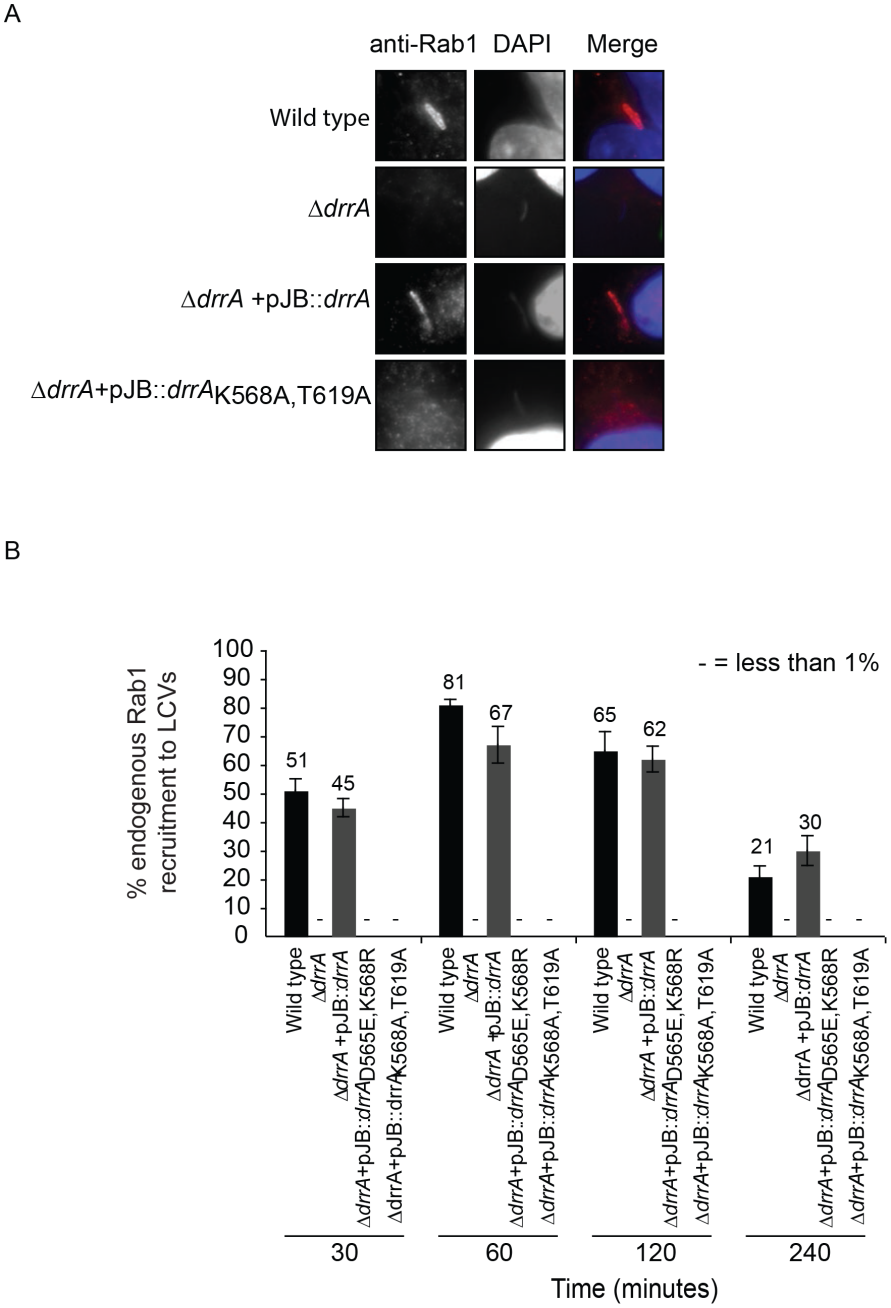
Targeting of DrrA by PI4P-binding is functionally important during *L. pneumophila* infection because it is required for localization of DrrA and Rab1 to *Legionella-*containing vacuoles. (A) Micrographs of HEK293 FcγR cells infected with various *Legionella pneumophila* Philadelphia-1 strains at 1 hour post-infection stained with Rab1b-specific antibodies and DAPI. Strains that do not produce DrrA (Δ*drrA*) or produce DrrA containing mutations that perturb PI4P-binding (Δ*drrA* +pJB*drrA* K568A/T619A) fail to show vacuolar Rab1b signals. (B) Quantification of Rab1b recruitment to LCVs with wild type, mutant and complemented *L. pneumophila* strains at various time points post-infection. Vacuoles were stained for endogenous Rab1 recruitment using Rab1b specific antibody. At each time point 150 vacuoles were counted per strain from three independent samples. Data shown are from one experiment but in at least two other independent experiments the same Rab1b recruitment phenotypes were observed. Numbers shown above bars indicate percent infected.

Importantly, DrrA was detected by immunofluorescence microscopy on vacuoles containing wild type *L. pneumophila* and on vacuoles containing a Δ*drrA* strain complemented with wild type *drrA* on a plasmid; however, DrrA staining was not observed on vacuoles containing the Δ*drrA* strain complemented with the mutant alleles *drrA*_D565E,K568R_ or *drrA*_K568R,T619A_ (Figure S8A). Rab1 staining was conducted after infection to determine whether a defect in association of DrrA with the vacuole correlates with a defect in Rab1 recruitment to the vacuole. At 1 hr post-infection, >60% of the vacuoles containing wild type *L. pneumophila* stained positive for Rab1 by a process that was dependent on Dot/Icm function and on DrrA function. Rab1 recruitment to vacuoles was restored when a plasmid-encoded allele of wild type *drrA* was used to complement the Δ*drrA* strain. By contrast, Rab1 recruitment was not detected on vacuoles when plasmids encoding the *drrA*_D565E/K568R_ or *drrA*_K568A/T619A_ alleles were used to complement the Δ*drrA* strain (Figure 4, Figure S8B). These substitutions confirm that PI4P-binding is required for DrrA function *in vivo*. Importantly, these substitutions in the LEPR of DrrA did not affect Rab1 binding activity or Dot/Icm-dependent delivery of DrrA into the host cytosol during infection (Figure S8C,D). Thus, DrrA-mediated recruitment of Rab1 to the LCV *in vivo* requires both the PI4P-binding activity and MIM functions mediated by the LEPR.

### Effectors with a LEPR localize to the early LCV

**Figure 5:**
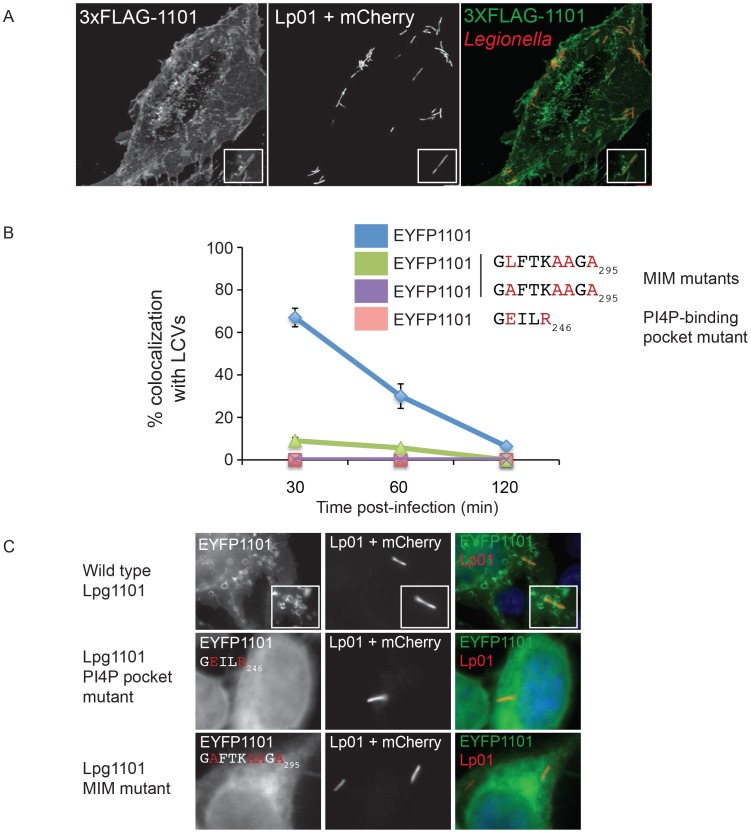
The effector Lpg1101 requires both its C-terminal LEPR and MIM regions for vacuolar localization. (A) Confocal micrograph of HEK293 cells stably expressing both FcγRII and 3XFLAG-tagged Lpg1101 and infected with wild type Lp01. The localization of 3xFLAG-tagged Lpg1101 was detected by indirect immunofluorescence using indirect the affinity purified anti-Lpg1101 antibody (1∶100) and secondary Alexa Fluor 488 (green). *L. pneumophila* were detected due to constitutive expression of mCherry (red). Lpg1101 was detected at both at the cell periphery and surrounding *Legionella*. (B) Quantification of the localization of EYFP-tagged Lpg1101 and LEPR and MIM mutant variants with intracellular Lp01 at 30, 60 and 120 min post-infection. Amino acid substitutions are shown in red. (C) Fluorescent micrographs of the localization of EYFP-Lpg1101 (EYFP1101) and the LEPR (D243E/K246R) and MIM (L288A/V292A/T293A/I295A) variants in cells infected with mCherry expressing *L. pneumophila* strain Lp01 (red). Hoechst 33343 was used to stain DNA (blue).

The presence of PI4P on vacuolar membranes and the shared targeting of the LEPR effectors to the plasma membrane suggest that Lpg1101 and Lpg2603 will target the early LCV after uptake by mammalian cells. To address this question, we first assessed LCV localization of LEPR-containing effectors expressed ectopically in host cells. Although PM localization was apparent in transfected cells, we did not detect robust localization of 3X-FLAG-DrrA or 3X-FLAG-Lpg2603 produced ectopically to LCVs containing wild type *L. pneumophila* (data not shown), however in cells producing 3X-FLAG-Lpg1101 we detected circumferential staining around vacuoles containing *L. pneumophila* at early time points post-infection and this staining dissipated as the LCVs matured (Figure 5). Similar staining patterns were observed in a stable HEK293 cell line producing 3X-FLAG-Lpg1101 (Figure 5A) and during transient transfection of HeLa cells with a plasmid producing YFP-Lpg1101 (Figure 5C). Using the strong localization signal of Lpg1101 on early LCVs, the importance of the PI4P-binding pocket and MIM regions for vacuolar targeting of Lpg1101 was examined (Figure 5B,C). Mutation of residues in both the PI4P-binding pocket and the MIM domain disrupted the localization of Lpg1101 to the LCV. These data confirm that the LEPR domain in Lpg1101 mediates effector localization to the early LCV.

Localization of Lpg1101 and Lpg2603 produced endogenously and translocated during *L. pneumophila* infection was examined by indirect immunofluorescence using antibodies generated against purified recombinant proteins (Figure S9). Immunoblot analysis demonstrated that these antibodies detected plasmid-encoded Lpg1101 and Lpg2603 produced from a constitutive promoter in *L. pneumophila* (Figure S9A), however, endogenous Lpg1101 was not detected in the infectious wild type *L. pneumophila* grown to early stationary phase. A limited number of Lpg1101-positive LCVs were observed 30 minutes after infection (Figure S9B), however, staining was weak and could not be confirmed to be specific. Thus, Lpg1101 may be produced at low levels or regulated differently than Lpg2603, and additional studies aimed at localization of Lpg1101 were not conducted.

**Figure 6:**
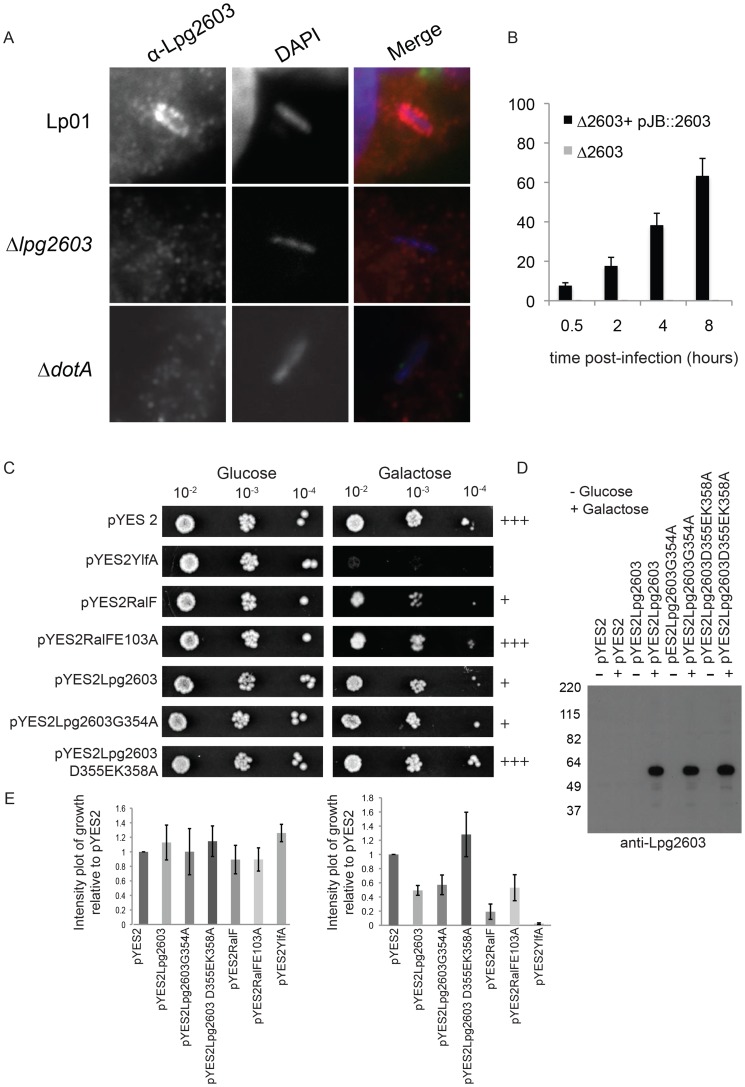
Lpg2603 localizes to LCVs and its intracellular function requires the LEPR domain. (A) Fluorescent micrographs showing colocalization of the effector Lpg2603, stained with affinity purified Lpg2603, and *L. pneumophila*, stained with DAPI, in bone marrow-derived macrophages from A/J mice at 3 hours post-infection. Colocalization was not observed in infections with the Δ*lpg2603* or Δ*dotA* deletion strains (lower panels). (B) Quantification of colocalization of Lpg2603 and intracellular *L. pneumophila* over early to mid phases of infection in HeLa cells. At each time point, 100 Hoechst 33342 stained bacteria were assessed for the presence or absence of lpg2603 for each strain, and data represent the average of results from three independent experiments. (C) Growth inhibition by ectopic production of Lpg2603 in yeast is restored by D355EK358A mutation within the PI4P-binding domain. Shown are 10-fold serial dilutions of *S. cerevisiae* harboring either vector alone (pYES2) or plasmids encoding YlfA, wild-type RalF protein, catalytically inactive RalF E103A mutant protein, Lpg2603, Lpg2603 mutants D355EK358A and G354A. Yeast plating efficiency on medium containing glucose was compared to medium containing galactose. (D) Immunoblot analysis (anti-Lpg2603) of cellular lysates from the indicated yeast strains shows equivalent levels of Lp2603 and mutants upon galactose induction. (E) Quantification of *S. cerevisiae* growth in non-inducing (glucose) and inducing conditions (galactose). Values are relative to vector only controls and were calculated using Image J software using two replicates for each condition.

Endogenously produced Lpg2603 was detected on LCVs containing wild type *L. pneumophila* and was not detected on vacuoles containing the Δ*dotA* mutant or the Δ*lpg2603* mutant (Figure 6A, Figure S9C). The percent of vacuoles that stained positive for Lpg2603 increased over time and maximal localization was observed on mature vacuoles containing replicating *L. pneumophila* (Figure 6B). Interestingly, infection of cells with *L. pneumophila* Δ*lpg2603* producing the mutant Lpg2603_K358A,T402A_ protein revealed that this LEPR mutant retains the ability to localize to the LCV at 6 hours post-infection (Figure S9C). Thus, endogenous Lpg2603 translocated during infection localizes to the LCV, however, localization of Lpg2603 to the mature LCV may not require PI4P binding mediated by the LEPR domain.

Although the function of Lpg2603 remains unknown, ectopic production of Lpg2603 in *Saccharomyces cerevisiae* interfered with yeast replication by a process that required a functional LEPR domain. A yeast growth defect was observed in cells expressing Lpg2603 and Lpg2603 G354A, which localize to the PM in mammalian cells, but not in yeast expressing the non-PM-targeting Lpg2603 D355E,K358R mutant (Figure 6C,E, Figure S3B). Similar levels of Lpg2603 expression were observed for all Lpg2603-expressing yeast strains (Figure 6D). The growth phenotype observed for Lpg2603 was weaker than that observed for RalF and YlfA, effectors previously found to cause growth defects in yeast. Taken together, these results suggest the PI4P-binding domain of Lpg2603 is important for its function inside eukaryotic cells.

### Binding of DrrA to the PM requires a PIP signature

**Figure 7:**
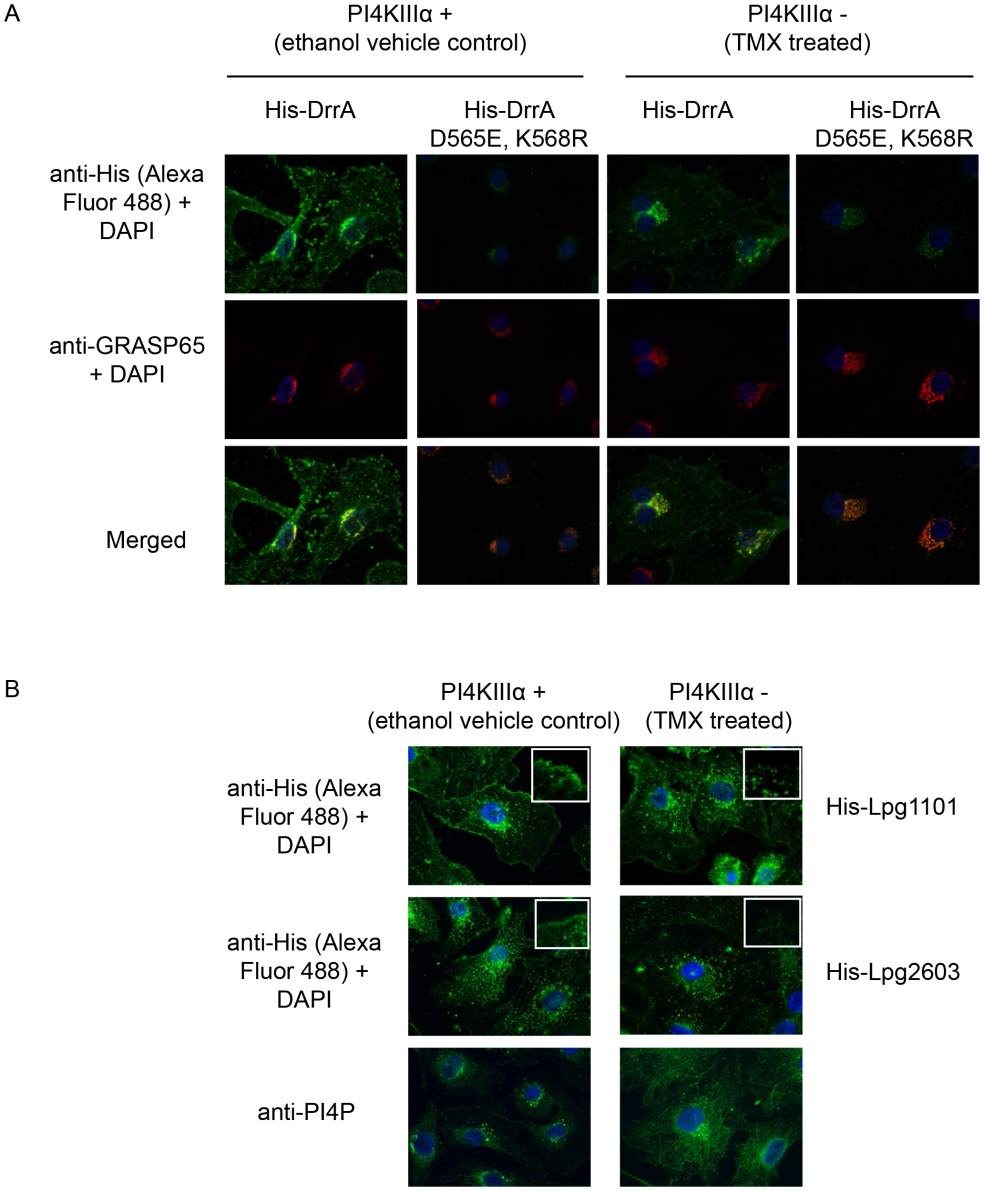
PI4KIIIα is important for localization of LEPR effectors to the PM. (A) Semi-intact assay showing the effects of deletion of PI4KIIIα on localization of DrrA. Full length purified His-DrrA or His-DrrA D565E,K568R (5 µg/well) was added to digitonin treated immortalized mouse embryonic fibroblasts (MEFs) containing (Z)-4-hydroxytamoxifen (TMX) inducible Cre-recombinase and f*lox*ed PI4KIIIα. Cells were either treated with ethanol (control) or TMX (1.5 µM) for 2 days and then used 7 days later to assess DrrA localization. After washing away unbound protein, cells were fixed with 4% PFA and His-tagged detected using a specific antibody at 1∶500 (green). GRASP65 (red) and DAPI (blue) staining were used to visualize the Golgi-apparatus and nucleus, respectively. (B) Semi-intact assay showing the effects of deletion of PI4KIIIα on localization of Lpg1101 and Lpg2603. Experiment performed as in (A) using 5 µg/well His-tagged Lpg1101 and Lpg2603. For reference, fluorescent images of PI4P staining with the anti-PI4P antibody from Echelon biosciences are shown in the lower panel.

A permeabilized cell system was used to study the localization of effectors with domains containing a LEPR (Figure S10). The binding of purified His-tagged LEPR effectors was assessed using HEK293 cells that had been permeabilized with digitonin, which allows recombinant proteins to gain access to the cytosolic surface of the PM and intracellular organelles. The His-LEPR effectors were found at the PM with some detectable Golgi staining in the permeabilized cells treated with His-DrrA. Peripheral localization was not observed using purified His-SteC, a *Salmonella* effector, or when His-DrrA mutant proteins that cannot bind PI4P were used (Figure S10A,B). Pretreatment of the cells with ionomycin, a calcium ionophore that stimulates hydrolysis of both PI4P and PI(4,5)P_2_ in membranes [54], decreased localization of His-LEPR effectors to the PM (Figure S10D). To further confirm the requirement for PM PI4P in LEPR effector targeting to the PM we utilized mouse embryonic fibroblasts (MEFs) derived from conditional *PI4KA* knockout mice. Treatment of these cells with (Z)-4-hydroxytamoxifen (TMX) causes *cre*-mediated deletion of *PI4KA*, which encodes PI4KIIIα, and perturbs PM PI4P levels [48]. Using our semi-intact assay we observed that TMX-treatment caused a decrease in the levels of His-DrrA observed at the PM, compared to control cells (Figure 7). A significant change in Golgi morphology, determined by GRASP65 staining, was not observed after deletion of *PI4KA*, which is consistent with previous results [48]. His-DrrA_D565E/K568R_, which failed to bind to PI4P in immobilized lipid arrays, showed weak localization to the Golgi-apparatus in the presence or absence of functional PI4KIIIα, likely due to Rab1-binding. Diminished PM localization of His-Lpg1101 and His-Lpg2603 was also observed in TMX treated compared to ethanol control MEFs from the conditional *PI4KA* knockout mice (Figure 7B). As a comparison, we assessed the localization of purified MBP-SidC_609–776_, which binds PI4P *in vitro* [35]. This protein showed perinuclear localization in semi-intact assays in HEK293 cells that was consistent with localization to the Golgi apparatus (Figure S11). To test whether targeting of MBP-SidC_609–776_ is influenced by PI4KIIIβ we used the inhibitor PIK93 at 1 µM concentration and observed a substantial reduction in the perinuclear localization of SidC_609–776_. The reported PIK93 IC50 values for PI3K, PI4KIIIα and PI4KIIIβ are 16 nM, 921–1620 nM, and 17.5–28 nM, respectively. A substantial change in the plasma membrane localization of the control MBP-Lpg2603_134–434_ was not observed after PIK93 treatment. These results further indicate that PI4P at the PM is an important signature that is recognized by the LEPR in DrrA and suggest PI4KIIIα is important for generating the PI4P that mediates DrrA targeting to the PM.

### Spatial control of DrrA function *in vivo* involves the activity of PI4P metabolizing enzymes regulating ER/PM contact sites

**Figure 8:**
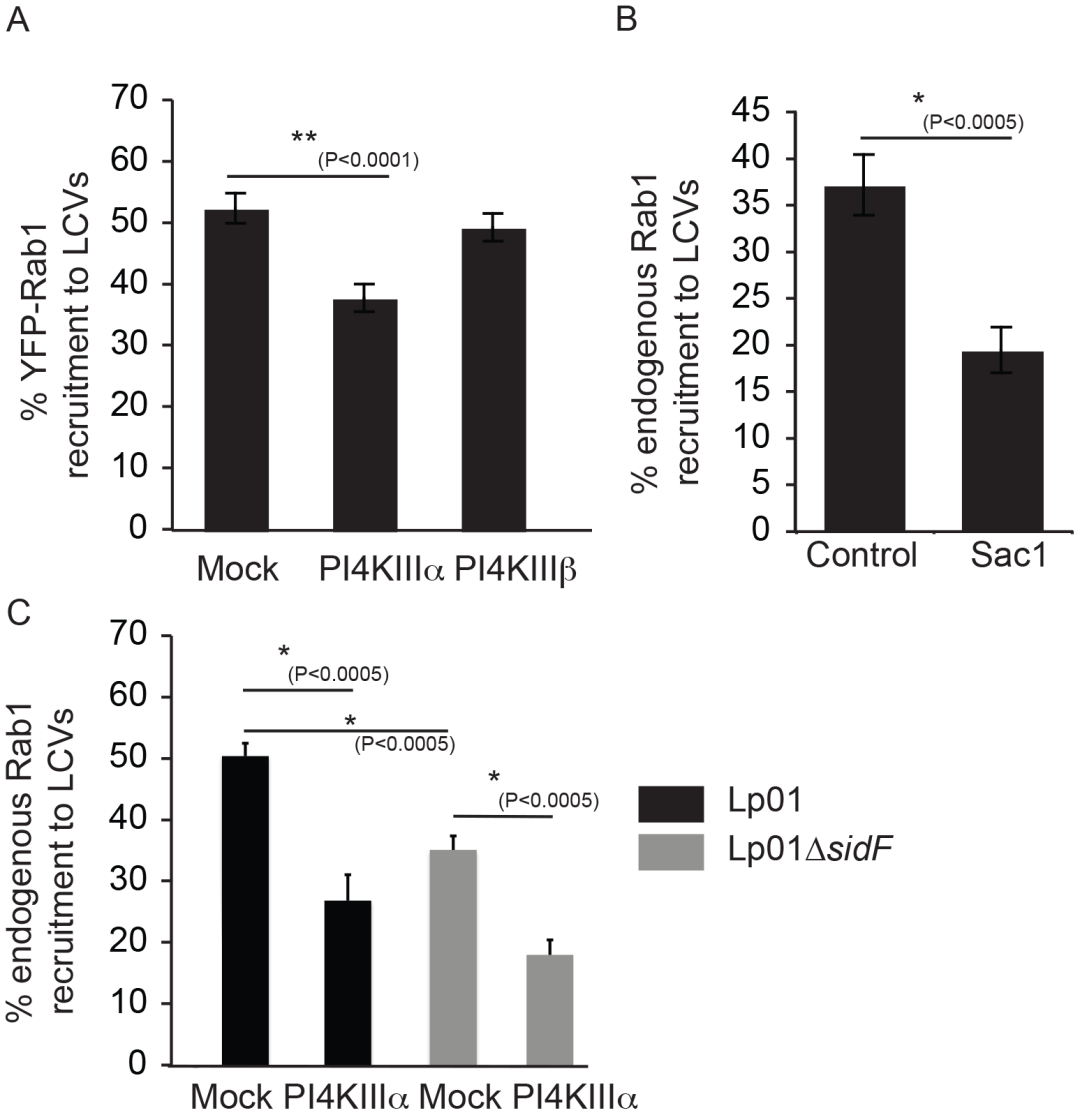
The host kinase PI4KIIIα is important for generation of a pool of PI4P used for DrrA targeting during infection. (A) Influence of knockdown of PI4KIIIα and PI4KIIIβ on DrrA-mediated recruitment of YFP-Rab1b to LCVs in HEK293 cells that stably express FcγR and YFP-Rab1b. After siRNA knockdown for 3 d, the HEK293 FcγR YFP-Rab1b cells were infected with wild type *L. pneumophila* expressing dsRed for 1 h before fixation. Quantification shown was obtained from at least 3 independent experiments with >300 vacuoles assessed for Rab1 recruitment for each condition. (B) Graph showing the percentage of Rab1b-positive LCVs formed by *L. pneumophila* strain Lp02+pAM239, expressing GFP, at 30 min post-infection in HEK293 FcγRII cells transiently expressing the human Sac1p phosphatase or the vector control. Data is from three independent experiments with >300 vacuoles assessed for Rab1 recruitment for each condition in each experiment. (C) Quantification of Rab1 colocalization with *L. pneumophila* wild type or Δ*sidF* strains, constitutively expressing GFP from plasmid pAM239C, at 60 min post infection in HEK293 FcγRII cells transfected with non-targeting (mock) or siRNAs targeting PI4KIIIα at 3 days prior to infection.

If DrrA recognizes a PI4P signature generated by PI4KIIIα, then reducing PI4KIIIα in the host cells would limit DrrA association with vacuoles containing *L. pneumophila* and interfere with Rab1 recruitment to the LCV. To test this hypothesis, siRNA was used to silence host PI4Ks and Rab1 recruitment to the LCV was measured. Silencing of PI4KIIIα resulted in a significant decrease in Rab1 recruitment to the LCV, whereas, silencing of PI4KIIIβ did not have a measurable effect on Rab1 recruitment to the LCV (Figure 8A). RT-PCR to measure transcript levels indicated that PI4KIIIα and PI4KIIIβ were silenced to similar levels in these cells; however, complete silencing was not obtained (Figure S12C), which could explain why Rab1 recruitment to the LCV in the PI4KIIIα-silenced cells was diminished but not eliminated. Because the lipid phosphatase activity displayed by the effector SidF generates PI4P through the hydrolysis of PI(3,4)P_2_ and PI(3,4,5)P_3_ a Δ*sidF* mutant was used to determine if this effector was important for generating the PI4P signature used for DrrA-mediated recruitment of Rab1 (Figure 8C). As reported previously, we found reduced SidC localization to the LCV in cells infected with the Δ*sidF* mutant compared to cells infected with the isogenic control strain (Figure S12A). Rab1 recruitment to the LCV was reduced in cells infected with the Δ*sidF* mutant, which would be consistent with SidF being involved in PI4P production on LCV. However, in Δ*sidF*-infected cells the levels of Rab1 recruitment to the LCV was even lower when PI4KIIIα was silenced. Thus, our data indicate that PI4KIIIα and the activity of SidF can independently produce a PI4P signature on the LCV that enables DrrA to recruit Rab1 to the PM-derived organelle.

Recent evidence from studies in yeast indicates that PI4P is metabolized at the PM through the coordinate functions of the PI4KIIIα protein and the ER-localized PI4P phosphatase Sac1 [51], [55]. Intriguingly, previous electron microscopy studies had noted a distinct similarity between ER-PM contact sites and the intimate association of ER-derived vesicles with the LCV [1], thus suggesting Sac1 on ER-derived membranes may play a role in consuming PI4P on the LCV in a manner similar to that used to control PI4P levels at the PM. Indeed, a significant reduction in the recruitment of Rab1 to the LCV was observed when the human Sac1 phosphatase was overproduced in HEK293 FcγRII cells by transient transfection (Figure 8B). These data suggest the enzymes that regulate PI4P levels at sites of interaction between the PM and ER regulate PI4P levels on the LCV, and the effector protein DrrA responds to changes in PI4P levels controlled by these enzymes.

### PI4KIIIα is important for *L. pneumophila* infection

**Figure 9:**
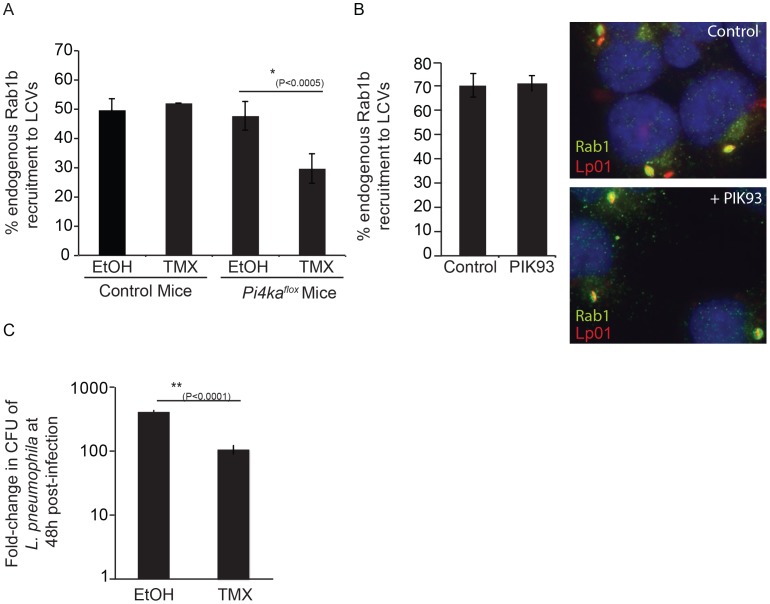
Influence of PI4KIIIα knockout on DrrA-mediated recruitment of Rab1b to LCVs in bone-marrow derived macrophages (*BMM*). (A) After treatment of *BMM* obtained from C57BL/6 or conditional *PI4KA^flox^* mice with either ethanol (EtOH) or (Z)-4-hydroxytamoxifen (TMX), which induces cre-mediated deletion of f*lox*ed PI4KIIIα, *BMM* were infected with wild type *L. pneumophila* expressing GFP for 1 h before fixation. Graph shows quantification of endogenous Rab1b recruitment to LCVs from three independent experiments with at least 150 vacuoles counted per treatment (EtOH/TMX) and mouse donor (C57BL/6/*PI4KA^flox^*) for each experiment. (B) Quantification of endogenous Rab1b recruitment to LCVs formed by Lp01+pAM239 in HEK293 FcγRII at 1 hour post-infection in cells treated with either DMSO (control) or 2 µM PIK93. Drugs were added to wells 30 min prior to infection and maintained throughout the subsequent steps. Data shown was obtained from 3 independent experiments with >200 vacuoles assessed for Rab1 recruitment for each condition per experiment. (C) Graph showing fold-difference in colony forming units (CFU) of *L. pneumophila* JR32Δ*flaA* at 48 hours post-infection compared to time zero. Results are from 18 wells from 2 inoculums per treatment (EtOH/TMX) and mouse donor (C57BL/6/*PI4KA^flox^*) for each time point from one experiment. No statistical differences were observed between the separate inoculums for each cell-type/treatment and results were averaged to give the final values. Error bars represent the SEM for fold-differences obtained for each condition. A decrease in *L. pneumophila* replication in TMX-treated *PI4KA^flox^* BMDM was also observed in two additional independent experiments.

Macrophages derived from the bone marrow of mice having a tamoxifen-inducible cre-mediated recombination system and an allele of *PI4KA* flanked by loxP sites (*PI4KA^flox^*) were used to further delineate the importance of PI4KIIIα during the intracellular lifecycle of *L. pneumophila*. Macrophages were treated for seven days with either tamoxifen to disrupt the *PI4KA* gene or with ethanol as a vehicle control and then infected with *L. pneumophila*. Macrophages were fixed 1 hr after infection and Rab1b staining was used to assess the function of DrrA. Immunoblot analysis and phosphoinositide measurement by HPLC revealed that tamoxifen-treated macrophages derived from the *PI4KA^flox^* mice had a reduction in the cellular levels of PI4KIIIα (Figure S12B, C). Consistent with siRNA results, Rab1b recruitment to the LCV was decreased significantly in macrophages from the *PI4KA^flox^* mice treated with tamoxifen compared to macrophages from the same mice treated with ethanol (Figure 9A, S12E). No defect in Rab1b recruitment to the LCV was observed in macrophages derived from control C57Bl/6 mice treated with either tamoxifen or ethanol. There was no defect in Rab1b recruitment to the LCV when PI4P levels were reduced through inhibition of PI4KIIIβ with PIK93 (Figure 9B). Because multiple effectors contain a LEPR and utilize PI4P for localization to the LCV, macrophages derived from the *PI4KA^flox^* mice were used to determine if disrupting the PI4P signature generated by PI4KIIIα might impact bacterial replication. These studies revealed a significant defect in *L. pneumophila* replication in tamoxifen-treated macrophages compared to the ethanol control-treated macrophages from the *PI4KA^flox^* mice (Figure 9C). Thus, generation of PI4P by PI4KIIIα is important for the spatial regulation of *L. pneumophila* effectors and disrupting PI4KIIIα has an adverse effect on replication of *L. pneumophila* in macrophages.

## Discussion

*L. pneumophila* must regulate the activity of at least 270 different effector proteins to successfully coordinate distinct stages of intracellular infection. Here we show that one mechanism used to control the localization of early effectors containing PI4P-binding domains is to utilize the host machinery at ER-PM contact sites that is involved in regulating PI4P-levels on the PM. Our data indicate that the host enzyme PI4KIIIα is involved in generating a pool of PI4P that mediates the localization of multiple effectors with a conserved PI4P-binding motif, and that the ER-localized Sac1 phosphatase is capable of consuming this PI4P to stimulate the dissociation of these effectors from the early LCV.

We report that multiple *L. pneumophila* Philadelphia-1 effectors utilize a conserved LEPR domain that targets these proteins to organelles derived from the plasma membrane. Previous studies have shown that the LEPR in DrrA binds to PI4P *in vitro* [23], and structural studies have identified a PI4P-binding pocket in this region [24], [25], [52]. These structural studies revealed a positively-charged pocket in the DrrA LEPR that mediates PI4P interactions, and a negatively-charged surface patch predicted to orient the protein for PI4P-binding. Many of these residues are conserved in the LEPR of Lpg1101 and Lpg2603, and these residues were critical for effector localization, as conservative mutations in key residues shared between these proteins abrogated both PM localization and *in vitro-*binding of these proteins to PI4P on immobilized lipid arrays. Thus, we conclude that the PI4P-binding activity displayed by these three effectors requires a functional LEPR that mediates PM localization of the effectors.

Targeting of DrrA to PM-derived LCVs was dependent on PI4P-binding, and directly correlated with the ability to recruit Rab1 to this membrane. Structural data identified K568 as key for interactions with the 4-phosphate of the bound ligand, and T619 as another key residue in the PI4P-binding pocket [25], [52]. The defect in localization of the DrrA K568A/T619A mutant to either the PM or LCVs during infection confirms the importance of PI4P-binding for the biological function of DrrA. These data supports a model whereby DrrA binds to the LCV through a process that requires recognition of PI4P on the vacuole membrane and then recruits Rab1 by stimulating GDP for GTP exchange [56].

Targeting of eukaryotic PI4P-binding proteins to specific cellular compartments is typically facilitated by interactions with other proteins. The proteins OSBP and FAPP are targeted to the Golgi apparatus through both PI4P-binding determinants and by interacting with the small GTPase Arf1 [42], [53]. Recent data on the dual interaction of FAPP1 with Arf1 and PI4P highlights the emerging concept of coincidence detection in cell signaling [57]. How effectors containing a LEPR preferentially recognize a PM-derived PI4P over another abundant pool of PI4P at the Golgi remains an interesting question. Although the LEPR in DrrA has been shown to interact with PM-localized syntaxin proteins [13], the proteins Lpg1101 and Lpg2603 did not show similar interactions, suggesting that host syntaxin proteins do not constitute a common determinant that facilitates localization of LEPR-containing effectors to the PM. It remains possible that there are other host proteins that bind to the LEPR that facilitate localization to the PM-derived organelle, or that there are other properties of this organelle that are sensed by the LEPR that confer specificity, such as membrane charge or lipid composition. One such determinant is the membrane insertion motif (MIM) region of DrrA [52].

Based on the structure of the C-terminus of DrrA in complex with dibutyl PI4P, helix 14 was predicted to penetrate phospholipid membranes. Successive mutations of four key exposed leucines in the MIM domain support this hypothesis as single L610A and L617A mutants showed a 4–6 fold reduction in affinity for PI4P-containing liposomes, and double (L614A/L615A) and triple (L610A/L614A/L615A) mutants showed 91 and 440-fold reduction in affinity, respectively [52]. Our data addressing the importance of these leucine residues for targeting of DrrA to the PM is consistent with the *in vitro* liposome affinity data. We observed a correlation between the decrease in affinity for PI4P-containing liposomes and the ability to target to the PM in eukaryotic cells. Despite a marked decrease in affinity of the single and double exposed leucine mutants for PI4P-containing liposomes, our *in vivo* data showed only the most severely affected triple and quadruple mutants failed to localize to the PM. This supports the importance of the strong PI4P-binding affinity of DrrA [24], [52]. Although the mutant L610A/L614A/L615A showed a shift in localization towards to cytoplasm, some peripheral localization was observed. PM association was not observed in the triple mutant (L614A/L615A/L617A) or in the quadruple mutant. Structural analysis places lysine 617 in direct contact with the acyl chains of the bound PI4P molecule, our data supports the importance of this lysine for membrane association because the phenotype for the L614A/L615A/L617A mutants is more severe than the L610A/L614A/L615A triple mutant. Substitutions in residues at conserved positions within Lpg1101 and Lpg2603 that are likely exposed MIM-domain residues, were also found to be important for plasma membrane localization of these effectors supporting the importance of the MIM-domain for membrane association. The effectors Lpg1101 and Lpg2603 do not show substantial localization to the Golgi-apparatus irrespective of the presence of the MIM domain, thus the MIM region is not the sole determinant that directs these effectors to the plasma membrane rather than the Golgi pool of PI4P. Although, the precise mechanism for specific targeting of the LEPR effectors to the plasma membrane pool of PI4P remains unknown it likely requires several signals as has been observed for specific membrane targeting of eukaryotic proteins to the plasma membrane [58].

Treating cells with PIK93, which is a potent and specific inhibitor of PI4KIIIβ, had no effect on DrrA function. Similarly, no effect on DrrA function was observed when PI4KIIIβ was silenced by siRNA. These data are in contrast to what has been observed in studies examining the localization of SidC to vacuoles containing *L. pneumophila* [23]. The effector SidC also binds membranes containing PI4P, however, the region of SidC containing the predicted PI4P binding determinant has no amino acid homology or predicted structural homology with the LEPR [34], [35]. This suggests that the mechanism of PI4P binding may be different. Consistent with this hypothesis, it has been shown that DrrA dissociates from the LCV shortly after bacterial replication begins, whereas, the protein SidC remains localized to the mature LCV [34]. When PI4KIIIβ levels were reduced in Drosophila Kc167 cells the amount of SidC associated with the LCV was reduced, suggesting a role for this host protein in mediating SidC localization [23]. The effector phosphatase SidF is one candidate bacterial protein that could maintain a pool of PI4P recognized by SidC during infection, which would be consistent with data showing that an LCV formed by the Δ*sidF* mutant displays reduced levels of SidC [36]. However, we found that the Δ*sidF* mutant also reduced vacuolar levels of Rab1 suggesting both PI4KIIIα and SidF function at early stages of infection to ensure that the LCV membrane has a PI4P signature. These data could indicate that LEPR effectors and SidC have different PI4P-binding modalities, but there is some overlap in the proteins used to generate and maintain the PI4P pools recognized by these effectors.

In addition to generation of vacuolar PI4P by host kinases and SidF, other mechanisms may further modulate PI on the *L. pneumophila* phagosome. Whether other effector proteins exist with PI-kinase or phosphatase activity is unknown, but the host phosphatase OCRL has been reported to localize to the *L. pneumophila* phagosome [59]. OCRL normally localizes to the Golgi-apparatus, endosomes and the PM (after specific stimulation) [60]. It possesses multiple protein-protein interaction domains as well as a PH domain, and is proposed to function in multiple membrane trafficking pathways, including between early endosomes and the Golgi and clathrin-mediated endocytosis [60]–[64]. The complexity of OCRL function is highlighted by the multiple defects observed in patients with Lowe's syndrome, which have mutations in OCRL. Association of OCRL with membranes occurs through interaction of active GTP-bound GTPases Rab1, Rab5, Rab6 and Rab8, with the minimal Rab-binding domain defined as amino acids 555–678 [65]. Although a mechanism for recruitment of OCRL to LCVs through activated Rab GTPases is possible, an N-terminal LVA (*Legionella* vacuole association) domain of OCRL, which includes amino acids 1–236, is sufficient for vacuolar association of OCRL. In *Dictyostelium discoideum* deletion of the homologue of human OCRL Dd5P4 was reported to show a number of phenotypes including reduced uptake, enhanced trafficking and replication, as well as a reduction in SidC recruitment to LCVs. This data suggests that the function of OCRL is somehow deleterious for *L. pneumophila*, but the molecular mechanisms behind these phenotypes are unknown. Clearly, vacuolar PI4P-levels must be tightly controlled during infection and mechanisms may be required to reduce vacuolar PI4P-levels or remove OCRL from vacuoles at later stages of infection. We found that the ER-localized PI4P phosphatase protein Sac1 interfered with the association of LEPR effectors with the LCV, supporting a role for these proteins in regulating the PI4P signal detected by the LEPR to signal when the PM-derived vacuoles have successfully been remodeled by ER-derived membranes. Alternatively, the role of OCRL on LCVs may not be simply PI4P production, but rather it may function in concert with its other binding partners to mediate other processes that influence vacuolar remodeling.

Pharmacological and genetic inhibitors indicated an important role for PI4KIIIα in generating PI4P used for localization of these effectors to the plasma membrane. These data provide support to previous studies indicating a role for PI4KIIIα in generating PI4P at the PM [40], [48], [66], [67]. Our finding that PI4KIIIα is important to generate the vacuolar PI4P recognized by the LEPR-containing effectors supports a role for PI4KIIIα at the PM, but our results cannot distinguish the subcellular localization of the kinase during *L. pneumophila* infection. Contact between cortical ER and the PM is reported to be less common in mammalian cells compared to yeast [68], however, although recruitment of the LEPR-containing proteins occurs before the LCV becomes positive for luminal ER proteins, ER-derived vesicles associate with the LCV shortly after uptake of the bacteria into the PM-derived phagosome [1]. It is also possible that PI4KIIIα is directly co-opted by *L. pneumophila* to enhance vacuolar PI4P levels.

Although we did not detect a defect in intracellular replication in strains lacking all three of the LEPR-containing effectors, this lack of growth defect is expected due to the redundant mechanisms utilized by *L. pneumophila* to survive within host cells. Our kinetic analysis suggests that Lpg1101 acts very early during infection whereas Lpg2603 may function at several stages of the infection process. The yeast growth defect observed upon expression of Lpg2603 is consistent with a previously reported yeast lethality phenotype for this effector, which may be due to interference with the yeast secretory pathway [69]. The lack of a growth defect for yeast expressing the Lpg2603 PI4P-binding mutant suggests PI4P-targeting by Lpg2603 is functionally important during infection. Determination of the host targets of these effectors will allow future analysis of the importance of PI4-binding in modulating the activities of these effectors.

These data suggest a model for spatial and temporal regulation of effectors with a LEPR region. Upon *L. pneumophila* uptake, LEPR-containing effectors would localize to the LCV membrane through interactions with PI4P generated at the PM by PI4KIIIα. For DrrA, this localization to the LCV membrane enables the effector to recruit and activate Rab1 on the pathogen-occupied organelle. Similarly, other LEPR-containing effectors are predicted to have effector activities that are spatially regulated on the early LCV. Shortly after formation, the LCV begins to recruit ER-derived vesicles and the lipid composition of the PM-derived organelle changes to become more similar to that of the host ER. Our data indicate that the presence of the Sac1 phosphatase on these ER-derived vesicles consumes the PI4P that is used for binding of LEPR-containing effectors, and provides a signal to indicate successful remodeling of the early LCV by ER-derived vesicles. This would lead to dissociation of LEPR-containing effectors from the LCV. When DrrA binding to the LCV is abrogated, the balance in Rab1 dynamics would be shifted greatly towards a pathway of Rab1 dissociation through the activity of the *L. pneumophila* GAP protein LepB, an effector that has a hydrophobic membrane spanning domain that anchors the protein to the LCV membrane throughout the infection cycle. Thus, these data highlight how spatial and temporal regulation of *L. pneumophila* effectors is a sophisticated process controlled by both intrinsic determinants such as PI4P-binding regions, and extrinsic determinants such as host factors that generate the organelle-specific signatures and the activity of other bacterial effectors that have additional determinants that mediate spatial regulation.

## Materials and Methods

### Bacterial strains and plasmids

Bacterial strains, plasmids and primers are described in supporting Text S1 (Tables S1–S3 in Text S1). *L. pneumophila* strains were grown on charcoal-yeast extract plates or in aces-buffered yeast extract broth as described previously [70]. All *L. pneumophila* mutant strains described were constructed in the *L. pneumophila* strain Lp01. Chromosomal deletion mutants were made using the plasmid pSR47S as described previously [71]. *L. pneumophila* Lp01 and mutant derivatives expressing the *lux* operon of *Photorhabdus luminescens* were created by triparental mating of *L. pneumophila* with the plasmid pSR47-*ahpC::lux* [72] and the *E. coli* Tra^+^ helper strain RK600. Deletion mutants were complemented with plasmid pJB1806M45, with either wild type or mutant LEPR-effectors. Complementation strains were confirmed by Western-blot analysis using antibodies specific to the protein encoded by the complementation construct. Site-directed mutants were made using complementary primers containing nucleotide substitutions to generate the desired amino acid changes. After a two-step PCR reaction to generate mutant plasmids, non-mutant template DNA was removed by Dpn1 digestion. DNA was transformed into *E. coli* DH5α, and substitutions confirmed by sequencing.

### Mice and cell lines

The HEK293 FcγRII GFP-Rab1 stable cell line was generated by transfection of pcDNA-GFPRab1 followed by selection of colonies using Zeocin (Invitrogen). The HEK293 FcγRII 3XFLAG-Lpg1101 stable cell line was generated in a similar manner to the GFP-Rab1 stable cell line using Zeocin selection at 400 µg/ml. HEK293 cells and stable variants were maintained as described previously [5]. HeLa cells were maintained in DMEM containing 10% FBS. HEK293 and HeLa cell transfections were performed using Lipofectamine 2000 (Invitrogen) according to the manufacturers instructions. C57BL/6 and A/J mice were purchased from Jackson Laboratories. The laboratory of Pietro De Camilli provided conditional PI4KIIIα knockout mice. Animals were maintained in accordance with the Yale University Institutional Animal Care and Use Committee (IACUC) under protocol 2010-07847 approved May 15, 2012. Yale IACUC is governed by applicable Federal and State regulations, including those of the Animal Welfare Act, Public Health Service, and the United States Department of Agriculture and is guided by the U.S. Government Principles for the Utilization and Care of Vertebrate Animals Used in Testing, Research and Training. Murine bone marrow-derived macrophages were obtained from mice as previously described [73], and used fresh or stored as described [74]. For conditional knockout experiments bone marrow-derived hematopoietic cells were induced for 3 to 4 days with L-cell supernatant before addition of the vehicle control ethanol or 3 µM (Z)-4-hydroxytamoxifen. Cells were supplemented on day 7 or 8 with 10 ml/10 cm dish of RPMI-1640 with 10% L-cell supernatant, 10% FBS and 1.5 µM tamoxifen. After 10-days cells were visually inspected to confirm morphological changes (enhanced size) associated with knockout, and seeded into 24-well plates in media without drugs or antibiotics for infection the following day. Knockout was assessed by western-blot analysis and phosphoinositide analysis.

### Infection of HEK293 FcγRII cells and preparation of microscopy samples

HEK293 FcγII cells were seeded onto poly-L-lysine (MW 75,000–150,000; Sigma Aldrich) treated coverslips 24 h prior to infection with opsonized bacteria at a multiplicity of infection (moi) of 2. The bacteria were pre-complexed with a rabbit anti-*Legionella* antibody (1∶1000 or 1∶2000) at 37°C for 20 min prior to infection. Studies assessing localization of DrrA and Rab1 were assessed a times indicated in figure legends or at 1 hour post-infection. Infections were synchronized by spinning cells at 1000 rpm for 5 min, incubating for 30 min at 37°C and then washing three times with warm PBS. Cells were fixed with 4% PFA for 10 min at 37°C. To ensure only internalized bacteria were analyzed for recruitment of DrrA or Rab1, external *L. pneumophila* were stained with rabbit Alexa Fluor 488 (1∶3000) and then washed extensively before cells were permeabilized using cold methanol. After antibody staining with antibodies against effector proteins or host-proteins and secondary Alexa Fluor 594 or Rhodamine Red-X antibodies, 4,6-diamidino-2-phenylindole staining (DAPI) was used to identify all bacteria. In experiments using the HEK293 FcγII YFP-Rab1 stable cell line infections were performed with dsRed expressing *L. pneumophila* strains and inside/out staining was not performed. For infection experiments assessing the effect of transiently expressed Sac1 on Rab1 recruitment to LCVs, Rab1 was detected by indirect immunofluorescence and the percentage of FLAG-Sac1 transfected cells was assessed in each experiment by staining separate coverslips with an anti-FLAG antibody. Only experiments that achieved at least 75% transfection of Sac1 were used to assess Rab1 recruitment. Analysis of endogenous Rab1 recruitment to vacuoles formed by wild type versus Δ*sidF* mutants was performed using bacteria constitutively expressing GFP from plasmid pAM239C, and Rab1 was detected by indirect immunofluorescence. In all experiments coverslips were mounted using the ProLong gold antifade reagent (Invitrogen).

### Infection of bone marrow-derived macrophages (*BMM*)

For infections designed to assess localization of proteins during infection of macrophages, *BMM*s were infected with *L. pneumophila* strains expressing GFP at a multiplicity of infection of 2 or 20 using the synchronized infection protocol described earlier and then incubated at 37°C for 1 hour. Cells were fixed and stained as described for HEK293 cells, except that inside/out staining was not performed. For growth assays, infections were performed as described by Zamboni *et al.* (2006), using an moi of 0.01 [75]. The fold differences were determined by dividing the values obtained at 48 h by the values obtained on day 0. *Ex vivo* growth assays were repeated at least three times, and the results obtained were similar. For *BMM* experiments using A/J mice *L. pneumophila* Lp01-derived strains were used. For experiments using C57BL/6 and *PI4KA^flox^ BMM*, infections were performed using *L. pneumophila* JR32Δ*flaA* [76].

### Yeast growth assays

Lpg2603 was cloned into pYES2 (Invitrogen) and substitution mutants created by site-directed mutagenesis. After sequencing, plasmids were introduced into competent *S.cerevisiae*. Overnight liquid YPD cultures were pelleted and washed once with water before being diluted to an OD 600 nm of 1. Serial dilutions were performed and 3 µl per dilution spotted onto either glucose or galatose/raffinose plates. After 3 days incubation plates were examined for yeast growth. Quantification of growth was performed using Image J. For yeast protein expression analysis yeast strains grown on YPD plates were used to inoculate overnight (synthetic dextrose) SD broths with either glucose or galactose/raffinose. Lysates were prepared by the glass bead method in extraction buffer containing 100 mM DTT and 100 mM PMSF.

### Protein purification

Affinity purification of tagged proteins was conducted as described [8]. Proteins were dialyzed in buffer containing 50 mM Tris/HCl, pH 7.5, 150 mM NaCl, 1 mM DTT and 1 mM MgCl_2_ using dialysis cassettes (Slide-A-Lyzer, Thermo Scientific) with a 20 kDa molecular mass cut-off. Protein concentrations were assessed by absorbance at OD280 nm accounting for the extinction coefficient, and then visually confirmed by Coomassie blue staining.

### PIP-strip analysis

PIP strips and arrays were purchased from Echelon Biosciences. Proteins used for binding to membrane PIP strips were used at 1 µg/ml using the protocol suggested by Echelon Biosciences. For PIP array binding, purified His-tagged proteins were added at 30 nM, and essentially fatty acid free BSA was used as a blocking reagent.

### Semi-permeabilized cell assay and inhibitors

For the semi-permeabilized cell assays, cells were treated with digitonin as described [13]. After washing away the digitonin, 1 to 5 µg/ml purified proteins (diluted in 125 mM K(OAc), 2.5 mM Mg(OAc)_2_, 25 mM Hepes-KOH (pH 7.4), 1 mg/ml glucose, 1 mM DTT) were added to the wells and incubated with gentle agitation for 1 hour at room temperature. This was followed by 2 washes of 1 ml each to remove unbound protein. Coverslips were fixed in 4% PFA for 10 min and stained with appropriate antibodies.

### Antibodies

Rabbit polyclonal antibodies against GFP, calnexin and MBP were purchased from Invitrogen, Stressgen and New England Biolabs, respectively. Monoclonal antibodies to His and Flag were from Sigma Aldrich, and the GM130 antibody from BD Transduction laboratories. Rabbit polyclonal antibodies to Rab1A and Rab1B were purchased from Santa Cruz. Rabbit polyclonal antibodies to *L. pneumophila* and DrrA were described previously [8]. Polyclonal antibodies against Lpg1101 and Lpg2603 were produced at Cocalico Biologicals (Reamstown, PA) using affinity purified histidine-tagged proteins as antigen to immunize the rabbits. To affinity purify the antibodies raised against Lpg1101 and Lpg2603, 1 mg of the purified His-tagged proteins were run on an SDS-page gel in a single lane spanning the entire gel width. After transfer to nitrocellulose membrane, the portion of the membrane with the correct migration size for the effector was cut out and then incubated with 5 ml of anti-serum for 2 hours at 4°C. The membrane was washed 3 times with cold PBS for 10 minutes each and then cut into small pieces and transferred into a 15 ml Falcon tube. 500 µl of 0.1 M Glycine (pH 2.5) was added to the tube and the tube gently agitated for 10 minutes at 4°C. The recovered solution was neutralized with 200 µL of Tris pH 8.8, dialyzed in cold PBS, and finally tested for specificity and cross reactivity against bacterial and host cell lysates. The anti-p115 and anti-PI4KIIIα antibodies were gifted by S. Mukherjee and the laboratory of Pietro De Camilli, respectively. Two anti-PI4KIIIβ antibodies were used to check for a vacuolar PI4KIIIβ signal.

### Sub-cellular fractionation

HEK293 FcγII cells at 80% confluency in 6-well dishes were transfected with 1 µg plasmid DNA per well with Lipofectamine 2000 (Invitrogen). After 16–24 h, cells were collected by centrifugation at 800 *g*, washed once in PBS, and resuspended in 500 µl of buffer (0.25 M sucrose, 150 mM KCl, 20 mM Hepes-KOH, 2 mM EDTA, 1 mM PMSF, 1 mM DTT). Cells were homogenized mechanically by repeatedly passing the suspension though a 27-gauge needle. After centrifugation at 1 700 *g* for 5 min the supernatant fraction was centrifuged for 30 min at 100 000 *g* in a Sorvall RC M120GX Ultracentrifuge. The resulting supernatant fraction was collected, and the pellet washed once in PBS before resuspension in SDS-page buffer. Equivalent amounts of supernatant and membrane fractions were loaded onto polyacrylaminde gels for separation and immunoblotting.

### siRNA knockdown

siGENOME siRNA oligos were obtained from Dharmacon (Thermo Scientific). Oligo sequences of individual siRNAs are listed in supporting Text S1 (Table S4 in Text S1). HEK293 FcγII cells were seeded onto poly-L-lysine coated coverslips in 24-well format. After 24 h cells were transfected with Lipofectamine 2000 and pools of four individual silencing reagents (A, B, C and D, final 12.5 nM of each) and incubated for 72 hours before *L. pneumophila* infection. Knockdown efficiency was validated by quantitative RT-PCR using cDNA synthesized from total RNA extracted from cells at 72 h post-knockdown. Primers used for RT-PCR are listed in Text S1 (Table S5 in Text S1). Additional details are available in the Materials and Methods section of supporting Text S1.

### Phosphoinositide analysis

Phosphoinositide analysis was performed as described previously [77]. Data were shown as the mean ratio of PI4P to PI(4,5)P_2_ levels (peak areas), which represent the bulk of PIP2 and PIP respectively, from three independent experiments.

### Microscopy

Epifluorescence micrographs were taken using a TE2000 (Nikon) inverted microscope and this microscope was used for all counting experiments. Confocal micrographs showing localization of ectopically expressed effectors were taken using a LSM510 microscope (Zeiss) with a 100×/1.4 numerical aperture objective, or a Fluoview FV10i (Olympus) microscope with a 60×/1.35 numerical aperture objective.

### Statistical analysis

To statistically assess significance, calculations were performed using the paired Student's t-test (homoscedastic two-tailed, paired) using Excel software (Microsoft). In all graphs error bars represent standard error of the mean (SEM).

### Gene accession numbers

The genes described in this manuscript are *lpg1101*, *lpg2603* and *drrA* with Genbank accession numbers of YP_095134.1, YP_096608.1 and YP_096471.1, respectively.

## Supporting Information

Figure S1 **LEPR-containing proteins are Dot/Icm effectors that bind PM-derived organelles.** (A) Graph showing Cya-based assay for translocation of effector proteins into host cells. CHO FcγRII cells were infected with *L. pneumophila* strains wild type (grey bars) or Δ*dotA* (black bars) harboring plasmids expressing Cya fusion with the indicated proteins. One hour after infection cells were lysed and cAMP was extracted and quantified. Levels of cAMP were also determined for cells infected with wild-type *L. pneumophila* expressing Cya alone (pCya). Each bar represents the mean cAMP value obtained from triplicate wells +/− standard error of the mean (SEM). (B) Coomassie-stained SDS-page gel of proteins from an *in vitro* binding assay to assess role of the LEPR in effectors binding to Rab1. GST-Rab1 was bound to Glutathione beads and then incubated with His-tagged wild-type DrrA or Lpg1101. After washing away unbound protein, proteins were eluted from the GST-beads, boiled and then analyzed by Coomassie staining. Unlike DrrA, the LEPR containing Lpg1101 protein was unable to bind to GST-Rab1 at a level detectable by this assay. (C) Representative images of the localization of the various GFP-DrrA constructs in HEK293 FcγRII cells. Separate panels show endogenous staining of the Golgi maker GM130. DrrA containing the GEF and PI4P-binding domains (amino acids 201–647) localizes to both the Golgi and PM. However, the GEF domain alone (201–500) is sufficient for Golgi localization. The PI4P-binding domain of DrrA (501–647) shows predominantly PM localization. This region is also the minimal region found to bind to plasma-membrane syntaxins (see Figure S3), however without the PI4P-binding region (amino acids 451–545) PM targeting does not occur. (D) Confocal xy images of HEK293 FcγRII cells transfected with constructs expressing GFP-DrrA or EYFP-Lpg1101 or EYFP-Lpg2603 or mutant protein variants, and RFP-PALM. (E) Summary of GFP- or YFP-tagged truncation constructs tested for localization to the PM in HEK293 FcγRII cells. Unshaded (white) constructs did not show plasma membrane (PM) localization. Constructs shaded grey or black gave a PM signal. In grey are the minimal C-terminal domain constructs that gave a PM signal.
(TIF)

Figure S2 **Intracellular growth analysis of single and triple LEPR mutants.** Defects in intracellular replication of single or the triple LEPR mutants were not observed in macrophages or in amoeba. (A) Fold change in colony forming units over 72 hours of *L. pneumophila* strains in A/J bone marrow-derived macrophages with an MOI of 1. Results are from two independent experiments, with triplicate wells in each experiment. (B) Graph showing fold change in relative luminescence units (RLU) of *lux*-expressing *L. pneumophila* strains in THP-1 cells using a 96-well plate format. Results shown are from two independent experiments as indicated and represent the average of 8–12 wells per assay. (C) Fold change in colony forming units over 48 hours of *L. pneumophila* strains in *Acanthamoeba castellani* with an MOI of 1. Data represent the average from two independent experiments performed in triplicate.
(TIF)

Figure S3 **Lpg1101 and Lpg2603 are functionally distinct compared to DrrA.** Western-blot images showing co-immunoprecipitation of (A) GFP-tagged DrrA 200–500, 451–647, 501–647, 451–545 or 546–647, or (B) EYFP-tagged-Lpg1101 or EYFP-tagged 2603 proteins and FLAG-tagged SNARE proteins produced in HEK293 FcγRII cells. Interactions were examined after precipitation of the SNARE proteins from cells extracts using anti-FLAG agarose. The antibodies indicated to the right of each blot show protein levels in the blots of the lysate (2.5–4% of input) and blots of the immunoprecipitate (IP). (C) Representative fluorescent micrographs (100×) of CHO FcγRII cells co-transfected with EYFP-Rab1a and mRFP-Lpg1101 or mRFP-LPg2603. The blue fluorescence is from 4′,6-diamidino-2-phenylindole (DAPI) staining.
(TIF)

Figure S4 **The LEPR is important for localization to the PM.** (A) Table summarizing the localization of EYFP-Lpg2603 site-mutants assessed by epifluorescence microscopy in HEK293 FcγRII cells. (B) Examples from summary table A. Epifluorescent micrographs of CHO FcγRII cells transfected with EYFP-Lpg2603 and mutant derivatives G354A and D355E,K358R. Shown in red is phalloidin staining. Arrows indicate fluorescence overlap between peripheral actin (phalloidin) and Lpg2603. (C) Representative images of HEK293 cells expressing GFP-tagged DrrA constructs 61–647, 451–647 and the minimal PM localization region 501–647. Data shows the effect of single and double amino acid substitutions at positions 565 and 568 within DrrA.
(TIF)

Figure S5 **The MIM domain is important for PM-localization of DrrA.** Micrographs of confocal Z-stacks of ectopically expressed GFPDrrA_501–647_ and lysine mutant variants (L610A, L614/615A, L617A, L610/614/615A, L614/615/716A, L610/614/615/617A) in HEK293 cells. Cells were co-transfected with mTagRFPPALM.
(TIF)

Figure S6 **The MIM domain is important for PM-localization of Lpg1101.** Micrographs of confocal Z-stacks of ectopically expressed EYFPLpg1101 and alanine mutant variants (L610A, L614/615A, L617A, L610/614/615A, L614/615/716A, L610/614/615/617A, K246A/T297A) in HEK293 cells. Closed white arrows indicate areas of peripheral membrane localization.
(TIF)

Figure S7 **PI4P is present on the LCV.** (A) Fluorescent micrographs showing localization of FAPP1 PH domain containing GFP fusion proteins in HEK293 FcγR cells infected with dsRed expressing wild-type (Lp02) or Δ*dotA* (Lp03) *L. pneumophila* for 30 min. (B) Fluorescent micrographs showing localization of GFP-FAPP1 and GFP-FAPP1R18L in HEK293 FcγR cells infected with dsRED-expressing *L. pneumophila* for 45 min. Cells were semi-permeabilized before fixation as described in supplementary methods. We were unable to visualize endogenous PI4P on vacuoles using the anti-PI4P antibody (Echelon biosciences). (C) Uninfected HEK293 cells transfected with YFP- or GFP-tagged LEPR effectors and variants, and semi-permeabilized before fixation. Blue color is DAPI staining.
(TIF)

Figure S8 **The LEPR in DrrA is important for in vivo function.** (A) Micrographs of HEK293 FcγRII cells infected with various *Legionella pneumophila* Philadelphia-1 strains at 1 hour post-infection stained with DrrA-specific antibodies and DAPI. Strains that do not produce DrrA (Δ*drrA*) or produce DrrA containing mutations that perturb PI4P-binding (Δ*drrA* +pJB*drrA* K568A,T619A) fail to show vacuolar DrrA signals. (B) Micrographs of EYFP-Rab1 dynamics during infection with strains expressing WT DrrA or DrrA D565E,K568R visualized using spinning disc microscopy. YFP-Rab1a HEK293 FcγR cells were infected with *L. pneumophila* stained with CellTracker Orange, images of infection were obtained every 50 seconds. Gallery contains images of projected confocal stacks displayed at the specified timepoints post-infection. Both merge and YFP-Rab1 channels are displayed to facilitate comparison of Rab1 levels on the vacuole. White arrowheads indicate a bacterium that becomes internalized during the course of imaging. (C) *In vitro* binding assay to assess influence of the C-terminal mutations on binding of DrrA to Rab1. GST-Rab1S25N (dominant negative) and GST-Rab1Q70L (constitutive active) were bound to Glutathione beads and then incubated with His-tagged wild type or mutant DrrA. After washing away unbound protein, proteins were eluted from the GST-beads, boiled and then analyzed by Western-blotting. All DrrA proteins were able to bind to GST-Rab1S25N (GDP-locked) with similar levels. (D) Western-blots showing translocation of DrrA during infection is not inhibited by expression of DrrA from a plasmid *in trans* or by site-mutations within the C-terminal PI4P domain. DrrA present in the bacterial pellet and cellular debris versus cell lysate on hour after infection of HEK293 FcγRII cells was assessed using the DrrA specific antibody. The plasma membrane protein syntaxin 3 is shown as a loading control for lysates and the process of cell lysis and fractionation.
(TIF)

Figure S9 **Analysis of translocated Lpg1101 and Lpg2603 localization during infection using indirect immunofluorescence.** (A) Western-blot analysis of Lpg1101 and Lpg2603 production in wild type, mutant and complemented *L. pneumophila* strains. Antibodies raised against His-tagged Lpg1101 and Lpg2603 was used to probe lysates of *L. pneumophila* grown to late exponential/early stationary phase on CYE media. Levels of the bacterial heat shock protein 60 (Hsp60) are shown as a loading control. (B) Fluorescent micrograph of bone marrow-derived macrophage from an A/J mouse infected with wild type Lp01 at 30 minutes post-infection. DNA is stained with DAPI, and the localization of Lpg1101 assessed using an affinity purified anti-lpg1101 antibody. (C) Fluorescent micrographs of *L. pneumophila* at 8 hours post infection in HeLa cells. Rabbit anti-Lpg2603 (1∶300) staining is shown in red, and mouse anti-*Legionella* (1∶5000) is shown in green. The wild type, Δ*lpg2603* and complemented strains posses vacuoles containing replicating bacteria, whereas singles bacteria were found in the Δ*dotA* infected cells. Vacuolar localization of Lpg2603 was not observed in the Δ*dotA* or Δ*lpg2603* mutant strains, however both the complemented strains examined, irrespective of the PI4P-binding capacity of the Lpg2603 proteins produced, localized to vacuoles containing replicating bacteria.
(TIF)

Figure S10 **Semi-permeabilized cell system to examine LEPR domain localization.** (A) Epifluorescent images of HEK293 FcγRII cells treated with digitonin and then incubated with purified His-tagged proteins DrrA, Lpg1101 and Lpg2603 from *Legionella pneumophila* or with a non-LEPR containing bacterial protein (His-SteC from *Salmonella enterica* serover Typhimurium). After washing away non-bound protein, proteins were detected using an anti-His antibody. (B) Micrographs showing localization of HisDrrA D565E/K568R, and HisDrrA K568A/T619A in semi-permeabilized cell system as in (A). Note that purified DrrA, with mutations that perturb PI4P-binding, show Golgi-localization. This is consistent with targeting due to Rab1-binding (see Figure S1C). (C) Fractionation of HEK293 cells after addition of His-DrrA, HisDrrA D565E/K568R, K568A/T619A in the semi-permeabilized cell system. The lower blot shows detection of Syntaxin 3 (Stx3) as a membrane fraction control. (D) Representative micrographs showing results of semi-permeabilized assay to assess the influence of ionomycin on localization of purified His-DrrA, His-Lpg1101, and His-Lpg2603.
(TIF)

Figure S11 **SidC localizes to the Golgi-apparatus in a PI4KIIIβ-dependent manner.** Epifluorescent images of HEK293 FcγRII cells treated with digtonin and then incubated with purified MBP-Lpg2603_134–434_ or MBPSidC_609–776_. After washing away non-bound protein, proteins were detected using an anti-MBP antibody. When indicated cells were preincubated with 1 µM PIK93 for 30 min prior to digitonin treatment. PIK93 was also maintained throughout the protein binding step. PIK93 causes a change in the localization of the Golgi-localized PI4P-binding probe from SidC but not the PM-localized Lpg2603 probe.
(TIF)

Figure S12 **Controls for siRNA knockdown and cre-mediated deletion of PI4KIIIα in** ***Pi4ka^flox^*** **–derived cells.** (A) Quantification of SidC-recruitment to vacuoles formed by wild type or Δ*sidF* mutant *L. pneumophila* expressing GFP (+pAM239C) in HEK FcγRII cells after 1 hour of infection. Fluorescent micrographs show representative staining pattern of SidC used for analysis. (B) Anion-exchange HPLC of glycerol-inositol phosphates obtained from control and knockout BMM. Data shown are the mean ratio of PI4P to PI(4,5)P_2_ levels (peak areas), which represent the bulk of PIP2 and PIP respectively, from three independent experiments. (C) Western-blot showing protein levels of PI4KIIIα at day 7 after TMX (tamoxifen) treatment or EtOH (ethanol vehicle control) treatment. Also shown is the level of the protein p115. (D) The extent of knockdown of PI4KIIIα and PI4KIIIβ mRNA by siRNA in HEK293 FcγRII cells was assessed by RT-PCR on cDNA obtained from mRNA extracted from cells on day 3. For each sample, transcript levels of genes targeted for silencing were compared to transcript levels of GAPDH. Results shown are the average of three independent experiments, and are presented as the percent of transcript detected relative to mock controls. (E) Representative micrographs taken with an epifluorescent microscope using a 60× objective showing BMDM infected with GFP expressing *L. pneumophila*. Asterisks indicated LCVs scored positive for Rab1 recruitment.
(TIF)

Text S1 **Supplementary information including Tables S1–S5, and additional methods and references for supporting figures.** Tables S1–S3 list the bacterial strains, plasmids and primers used in this study, respectively. Table S4 provides the sequences of siRNAs and Table S5 lists primers used for RT-PCR.
(DOCX)
