# Supplementary figures and images for: The Machinery at Endoplasmic Reticulum-Plasma Membrane Contact Sites Contributes to Spatial Regulation of Multiple Legionella Effector Proteins
Source: PLoS Pathog. 2014 Jul 3;10(7):e1004222. doi: 10.1371/journal.ppat.1004222 (PMC4081824; doi:10.1371/journal.ppat.1004222)

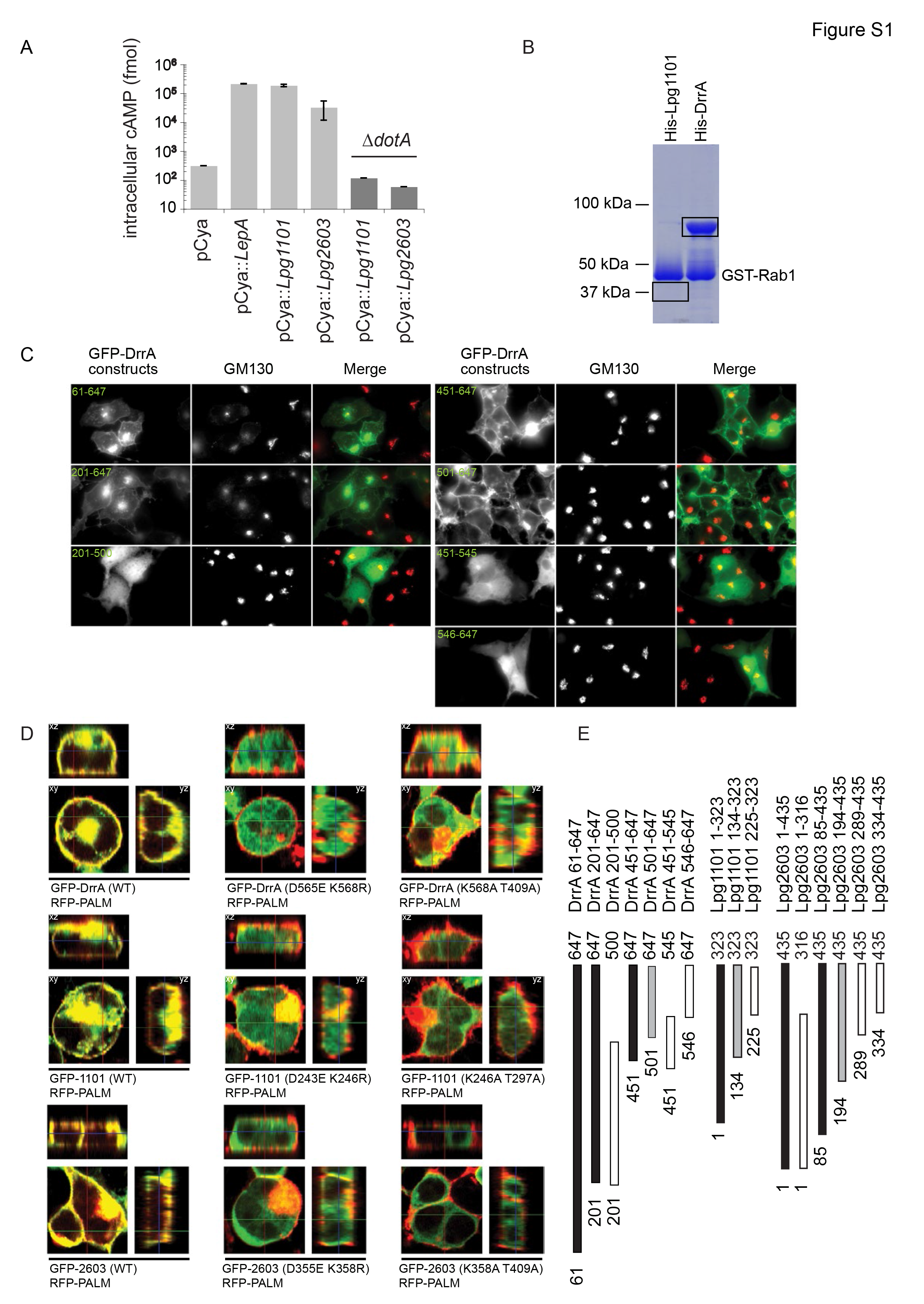

Supplement: Figure S1 — LEPR-containing proteins are Dot/Icm effectors that bind PM-derived organelles. (A) Graph showing Cya-based assay for translocation of effector proteins into host cells. CHO FcγRII cells were infected with L. pneumophila strains wild type (grey bars) or ΔdotA (black bars) harboring plasmids expressing Cya fusion with the indicated proteins. One hour after infection cells were lysed and cAMP was extracted and quantified. Levels of cAMP were also determined for cells infected with wild-type L. pneumophila expressing Cya alone (pCya). Each bar represents the mean cAMP value obtained from triplicate wells +/− standard error of the mean (SEM). (B) Coomassie-stained SDS-page gel of proteins from an in vitro binding assay to assess role of the LEPR in effectors binding to Rab1. GST-Rab1 was bound to Glutathione beads and then incubated with His-tagged wild-type DrrA or Lpg1101. After washing away unbound protein, proteins were eluted from the GST-beads, boiled and then analyzed by Coomassie staining. Unlike DrrA, the LEPR containing Lpg1101 protein was unable to bind to GST-Rab1 at a level detectable by this assay. (C) Representative images of the localization of the various GFP-DrrA constructs in HEK293 FcγRII cells. Separate panels show endogenous staining of the Golgi maker GM130. DrrA containing the GEF and PI4P-binding domains (amino acids 201–647) localizes to both the Golgi and PM. However, the GEF domain alone (201–500) is sufficient for Golgi localization. The PI4P-binding domain of DrrA (501–647) shows predominantly PM localization. This region is also the minimal region found to bind to plasma-membrane syntaxins (see Figure S3), however without the PI4P-binding region (amino acids 451–545) PM targeting does not occur. (D) Confocal xy images of HEK293 FcγRII cells transfected with constructs expressing GFP-DrrA or EYFP-Lpg1101 or EYFP-Lpg2603 or mutant protein variants, and RFP-PALM. (E) Summary of GFP- or YFP-tagged truncation constructs tested for localization [file ppat.1004222.s001.tif]

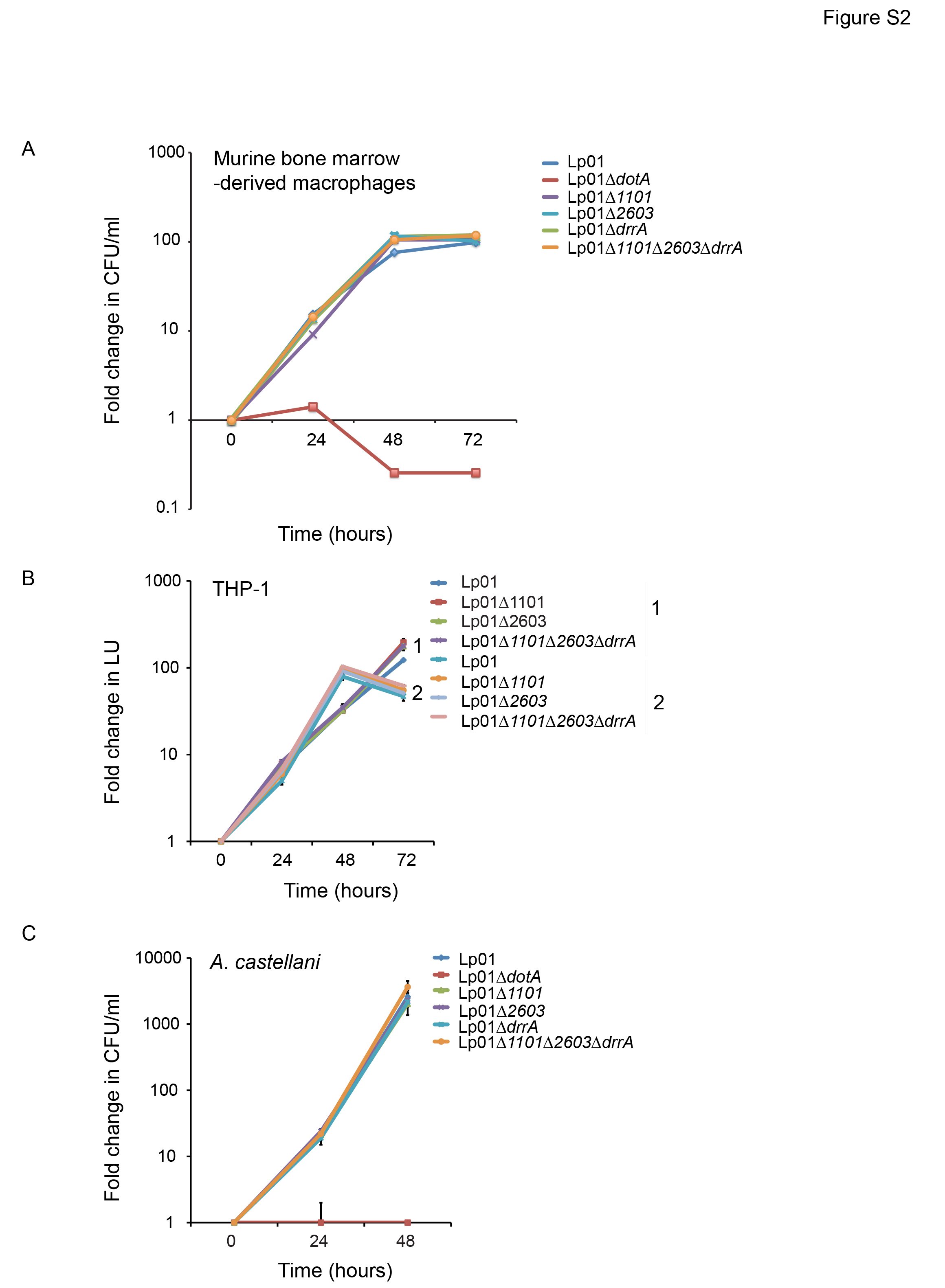

Supplement: Figure S2 — Intracellular growth analysis of single and triple LEPR mutants. Defects in intracellular replication of single or the triple LEPR mutants were not observed in macrophages or in amoeba. (A) Fold change in colony forming units over 72 hours of L. pneumophila strains in A/J bone marrow-derived macrophages with an MOI of 1. Results are from two independent experiments, with triplicate wells in each experiment. (B) Graph showing fold change in relative luminescence units (RLU) of lux-expressing L. pneumophila strains in THP-1 cells using a 96-well plate format. Results shown are from two independent experiments as indicated and represent the average of 8–12 wells per assay. (C) Fold change in colony forming units over 48 hours of L. pneumophila strains in Acanthamoeba castellani with an MOI of 1. Data represent the average from two independent experiments performed in triplicate. (TIF) [file ppat.1004222.s002.tif]

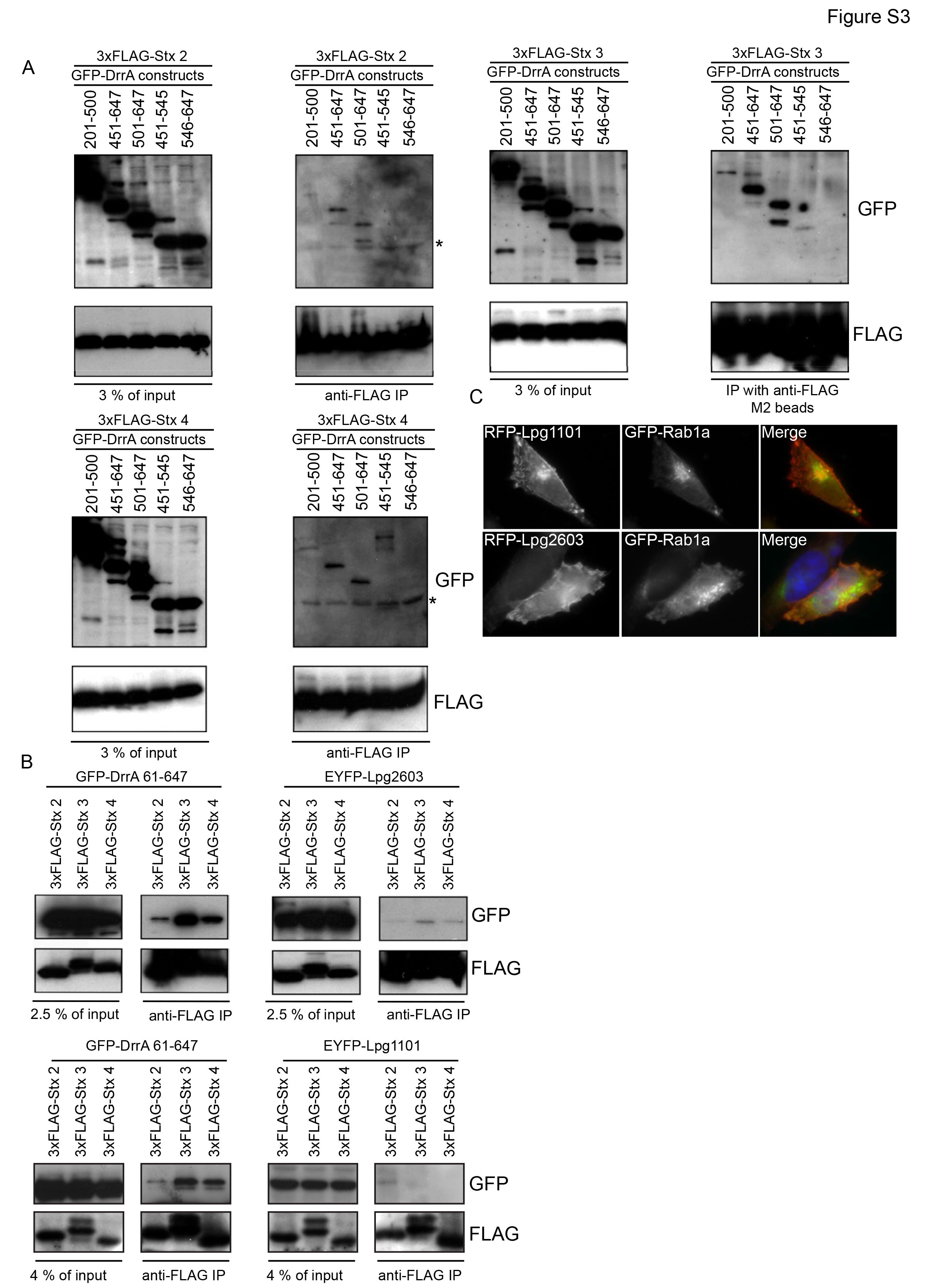

Supplement: Figure S3 — Lpg1101 and Lpg2603 are functionally distinct compared to DrrA. Western-blot images showing co-immunoprecipitation of (A) GFP-tagged DrrA 200–500, 451–647, 501–647, 451–545 or 546–647, or (B) EYFP-tagged-Lpg1101 or EYFP-tagged 2603 proteins and FLAG-tagged SNARE proteins produced in HEK293 FcγRII cells. Interactions were examined after precipitation of the SNARE proteins from cells extracts using anti-FLAG agarose. The antibodies indicated to the right of each blot show protein levels in the blots of the lysate (2.5–4% of input) and blots of the immunoprecipitate (IP). (C) Representative fluorescent micrographs (100×) of CHO FcγRII cells co-transfected with EYFP-Rab1a and mRFP-Lpg1101 or mRFP-LPg2603. The blue fluorescence is from 4′,6-diamidino-2-phenylindole (DAPI) staining. (TIF) [file ppat.1004222.s003.tif]

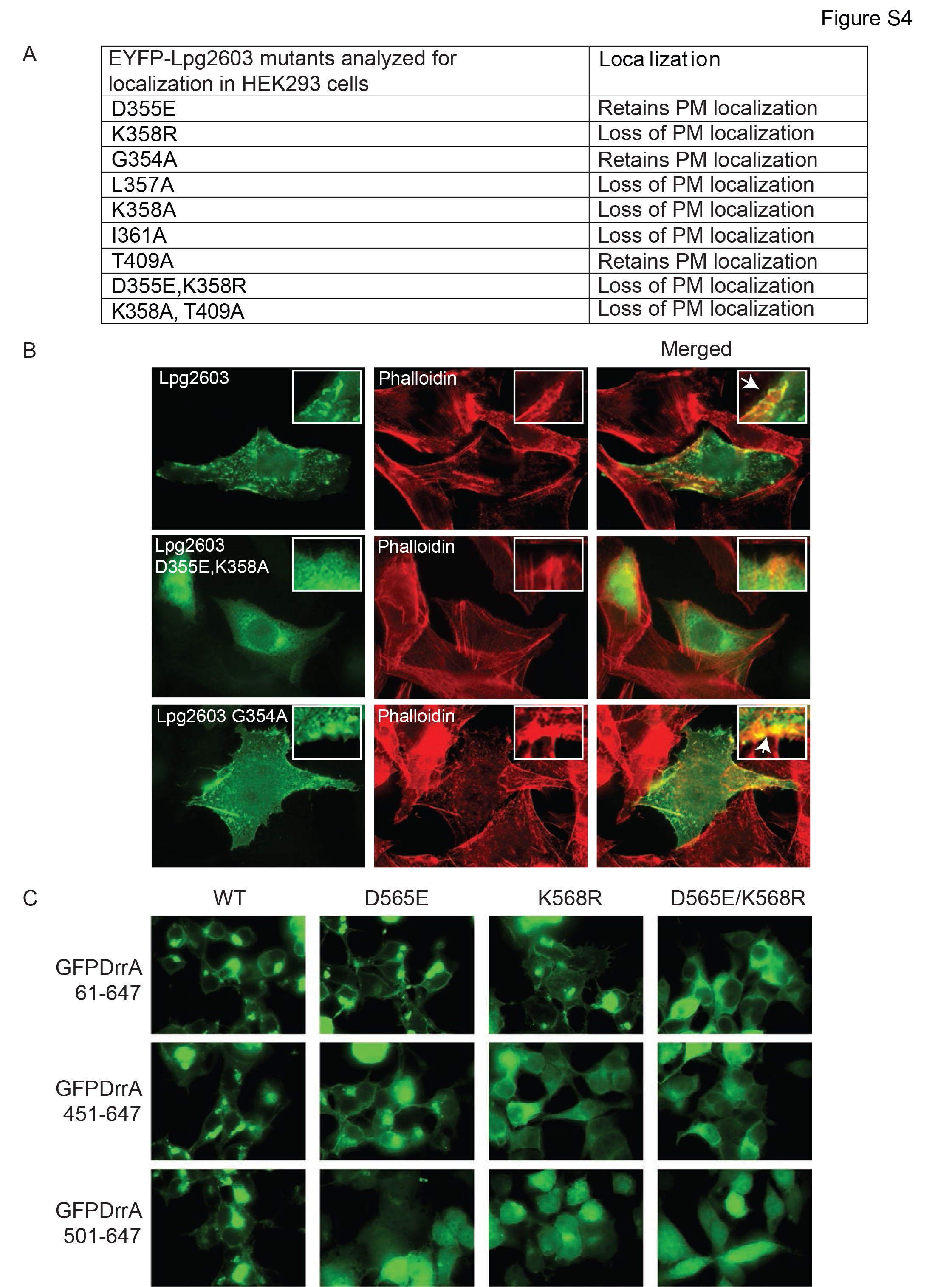

Supplement: Figure S4 — The LEPR is important for localization to the PM. (A) Table summarizing the localization of EYFP-Lpg2603 site-mutants assessed by epifluorescence microscopy in HEK293 FcγRII cells. (B) Examples from summary table A. Epifluorescent micrographs of CHO FcγRII cells transfected with EYFP-Lpg2603 and mutant derivatives G354A and D355E,K358R. Shown in red is phalloidin staining. Arrows indicate fluorescence overlap between peripheral actin (phalloidin) and Lpg2603. (C) Representative images of HEK293 cells expressing GFP-tagged DrrA constructs 61–647, 451–647 and the minimal PM localization region 501–647. Data shows the effect of single and double amino acid substitutions at positions 565 and 568 within DrrA. (TIF) [file ppat.1004222.s004.tif]

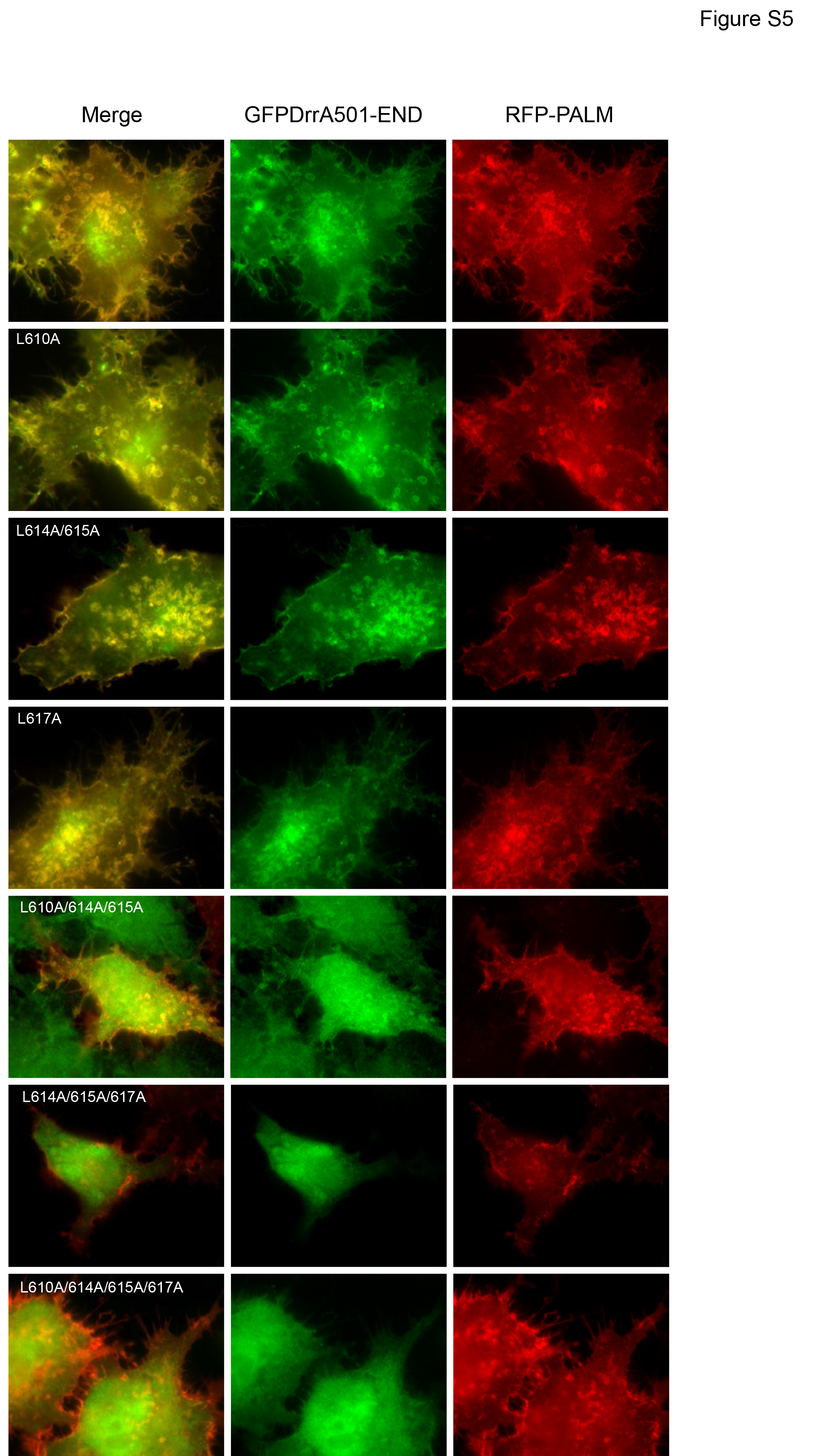

Supplement: Figure S5 — The MIM domain is important for PM-localization of DrrA. Micrographs of confocal Z-stacks of ectopically expressed GFPDrrA501–647 and lysine mutant variants (L610A, L614/615A, L617A, L610/614/615A, L614/615/716A, L610/614/615/617A) in HEK293 cells. Cells were co-transfected with mTagRFPPALM. (TIF) [file ppat.1004222.s005.tif]

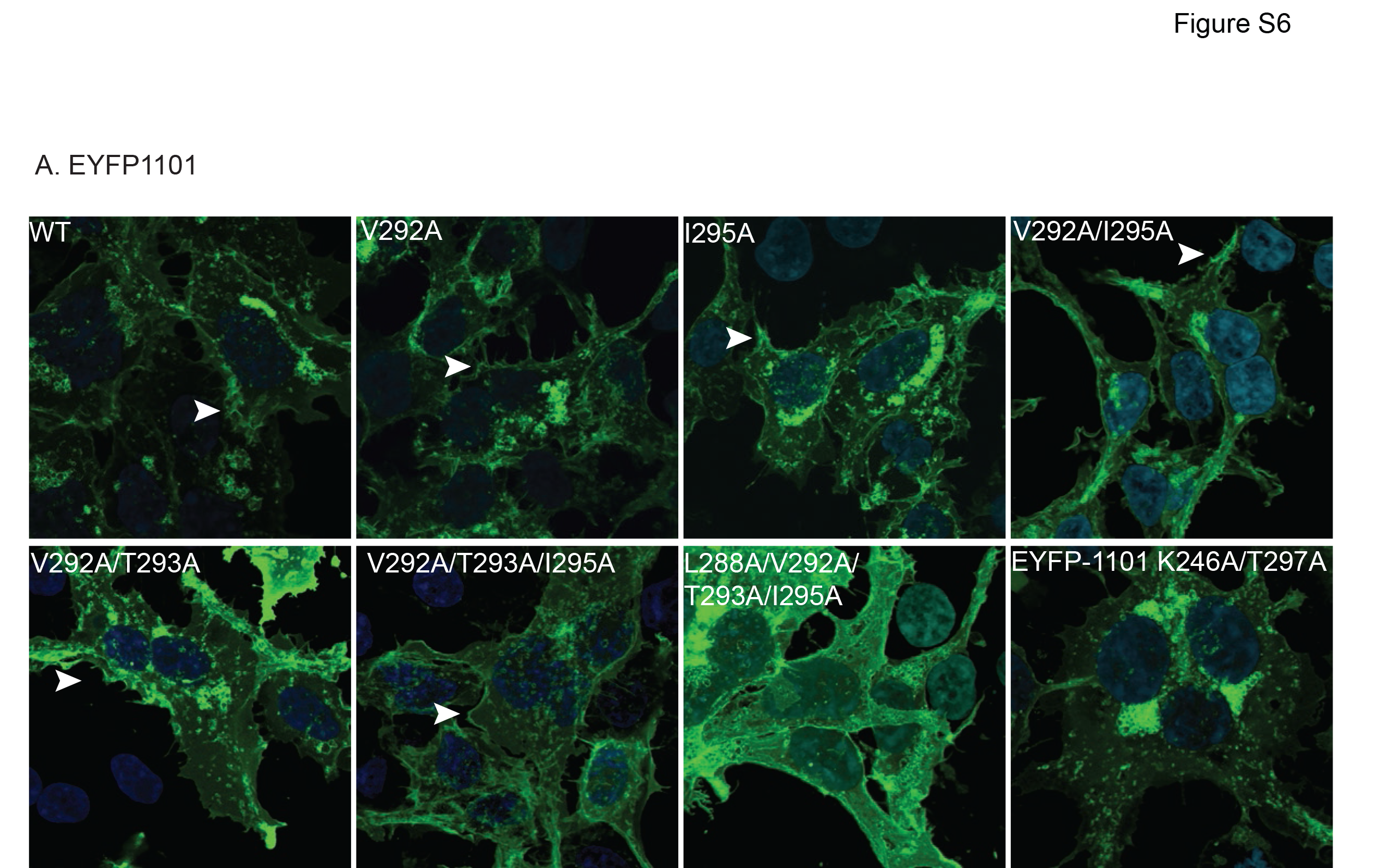

Supplement: Figure S6 — The MIM domain is important for PM-localization of Lpg1101. Micrographs of confocal Z-stacks of ectopically expressed EYFPLpg1101 and alanine mutant variants (L610A, L614/615A, L617A, L610/614/615A, L614/615/716A, L610/614/615/617A, K246A/T297A) in HEK293 cells. Closed white arrows indicate areas of peripheral membrane localization. (TIF) [file ppat.1004222.s006.tif]

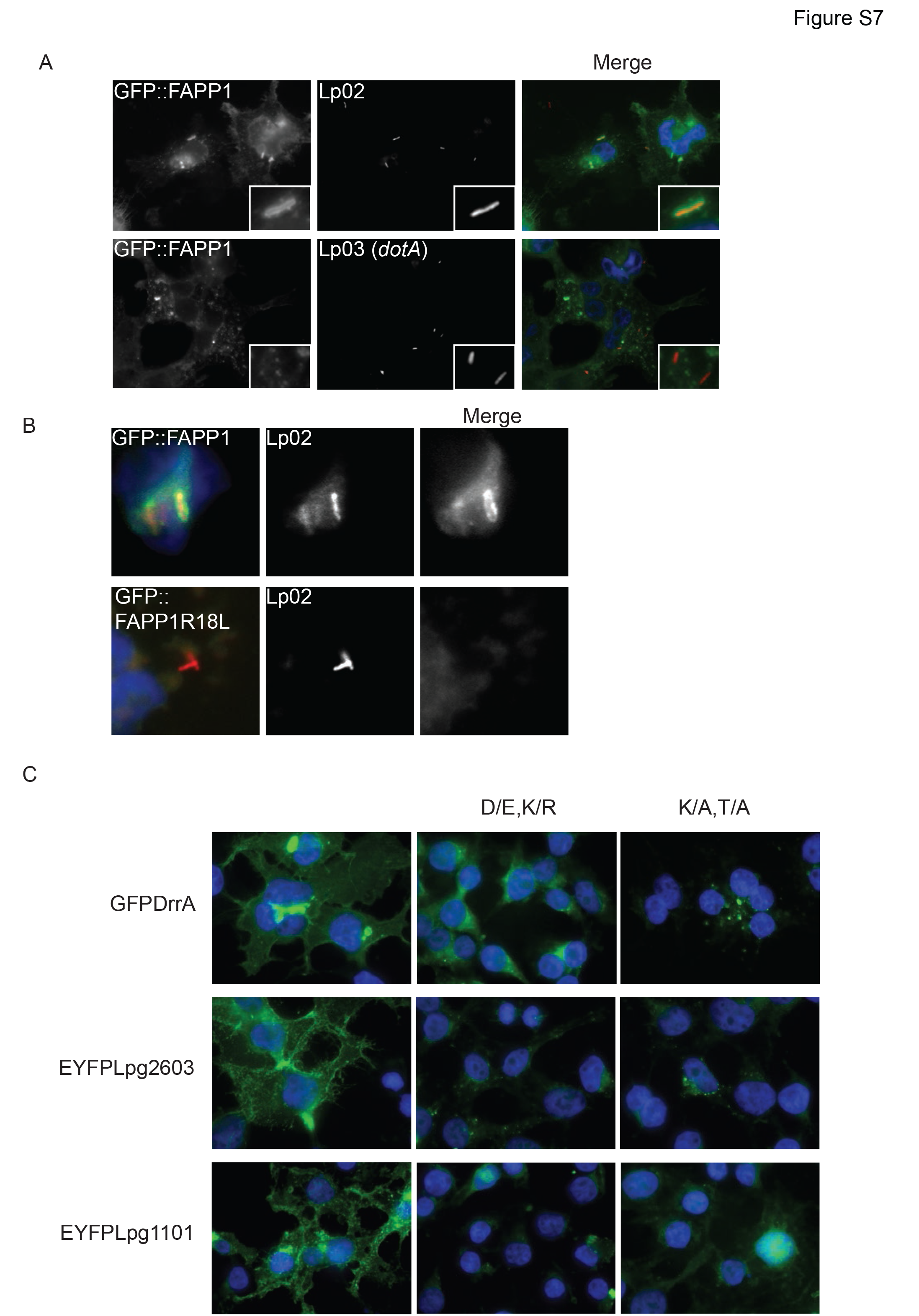

Supplement: Figure S7 — PI4P is present on the LCV. (A) Fluorescent micrographs showing localization of FAPP1 PH domain containing GFP fusion proteins in HEK293 FcγR cells infected with dsRed expressing wild-type (Lp02) or ΔdotA (Lp03) L. pneumophila for 30 min. (B) Fluorescent micrographs showing localization of GFP-FAPP1 and GFP-FAPP1R18L in HEK293 FcγR cells infected with dsRED-expressing L. pneumophila for 45 min. Cells were semi-permeabilized before fixation as described in supplementary methods. We were unable to visualize endogenous PI4P on vacuoles using the anti-PI4P antibody (Echelon biosciences). (C) Uninfected HEK293 cells transfected with YFP- or GFP-tagged LEPR effectors and variants, and semi-permeabilized before fixation. Blue color is DAPI staining. (TIF) [file ppat.1004222.s007.tif]

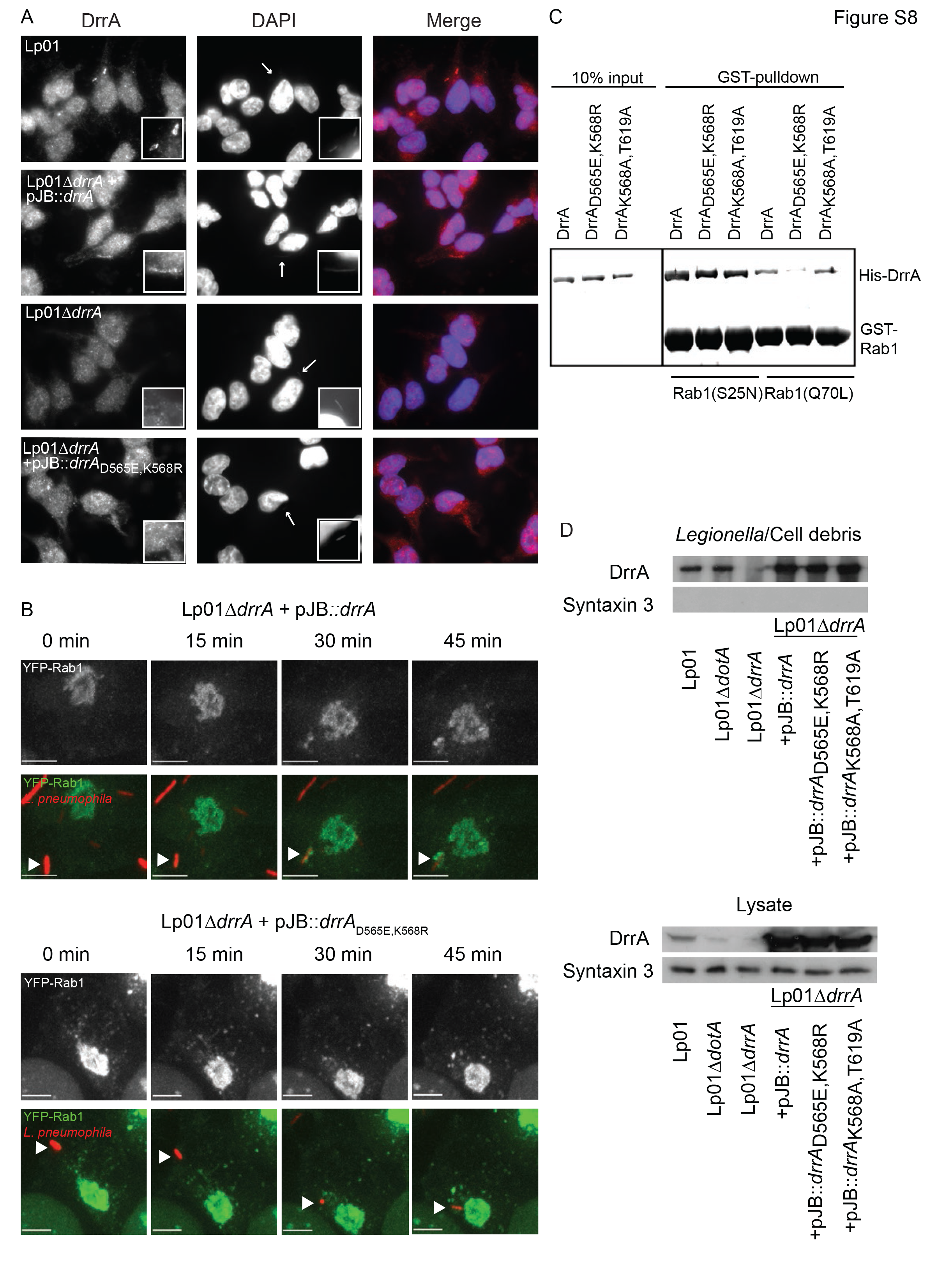

Supplement: Figure S8 — The LEPR in DrrA is important for in vivo function. (A) Micrographs of HEK293 FcγRII cells infected with various Legionella pneumophila Philadelphia-1 strains at 1 hour post-infection stained with DrrA-specific antibodies and DAPI. Strains that do not produce DrrA (ΔdrrA) or produce DrrA containing mutations that perturb PI4P-binding (ΔdrrA +pJBdrrA K568A,T619A) fail to show vacuolar DrrA signals. (B) Micrographs of EYFP-Rab1 dynamics during infection with strains expressing WT DrrA or DrrA D565E,K568R visualized using spinning disc microscopy. YFP-Rab1a HEK293 FcγR cells were infected with L. pneumophila stained with CellTracker Orange, images of infection were obtained every 50 seconds. Gallery contains images of projected confocal stacks displayed at the specified timepoints post-infection. Both merge and YFP-Rab1 channels are displayed to facilitate comparison of Rab1 levels on the vacuole. White arrowheads indicate a bacterium that becomes internalized during the course of imaging. (C) In vitro binding assay to assess influence of the C-terminal mutations on binding of DrrA to Rab1. GST-Rab1S25N (dominant negative) and GST-Rab1Q70L (constitutive active) were bound to Glutathione beads and then incubated with His-tagged wild type or mutant DrrA. After washing away unbound protein, proteins were eluted from the GST-beads, boiled and then analyzed by Western-blotting. All DrrA proteins were able to bind to GST-Rab1S25N (GDP-locked) with similar levels. (D) Western-blots showing translocation of DrrA during infection is not inhibited by expression of DrrA from a plasmid in trans or by site-mutations within the C-terminal PI4P domain. DrrA present in the bacterial pellet and cellular debris versus cell lysate on hour after infection of HEK293 FcγRII cells was assessed using the DrrA specific antibody. The plasma membrane protein syntaxin 3 is shown as a loading control for lysates and the process of cell lysis and fractionation. (TIF) [file ppat.1004222.s008.tif]

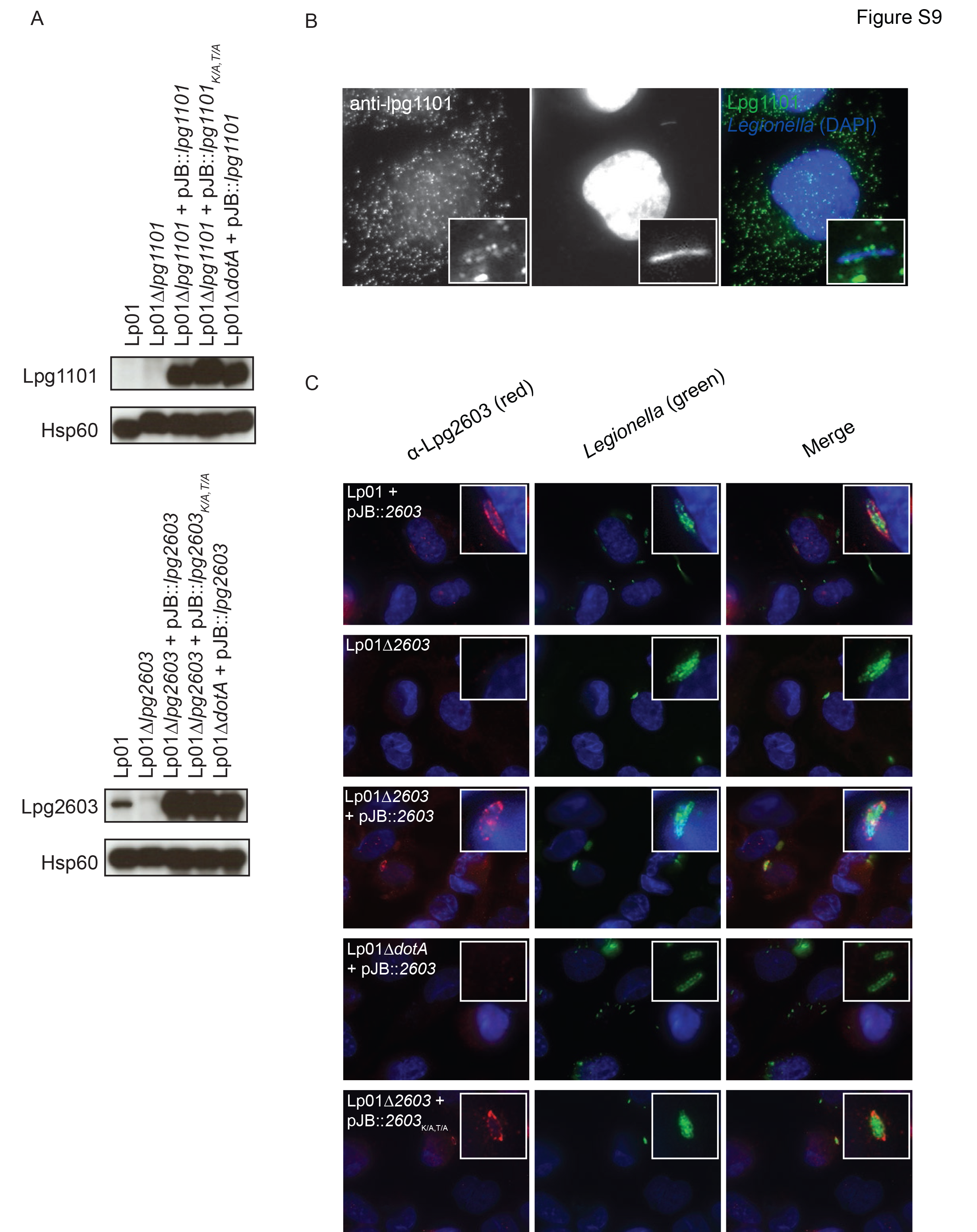

Supplement: Figure S9 — Analysis of translocated Lpg1101 and Lpg2603 localization during infection using indirect immunofluorescence. (A) Western-blot analysis of Lpg1101 and Lpg2603 production in wild type, mutant and complemented L. pneumophila strains. Antibodies raised against His-tagged Lpg1101 and Lpg2603 was used to probe lysates of L. pneumophila grown to late exponential/early stationary phase on CYE media. Levels of the bacterial heat shock protein 60 (Hsp60) are shown as a loading control. (B) Fluorescent micrograph of bone marrow-derived macrophage from an A/J mouse infected with wild type Lp01 at 30 minutes post-infection. DNA is stained with DAPI, and the localization of Lpg1101 assessed using an affinity purified anti-lpg1101 antibody. (C) Fluorescent micrographs of L. pneumophila at 8 hours post infection in HeLa cells. Rabbit anti-Lpg2603 (1∶300) staining is shown in red, and mouse anti-Legionella (1∶5000) is shown in green. The wild type, Δlpg2603 and complemented strains posses vacuoles containing replicating bacteria, whereas singles bacteria were found in the ΔdotA infected cells. Vacuolar localization of Lpg2603 was not observed in the ΔdotA or Δlpg2603 mutant strains, however both the complemented strains examined, irrespective of the PI4P-binding capacity of the Lpg2603 proteins produced, localized to vacuoles containing replicating bacteria. (TIF) [file ppat.1004222.s009.tif]

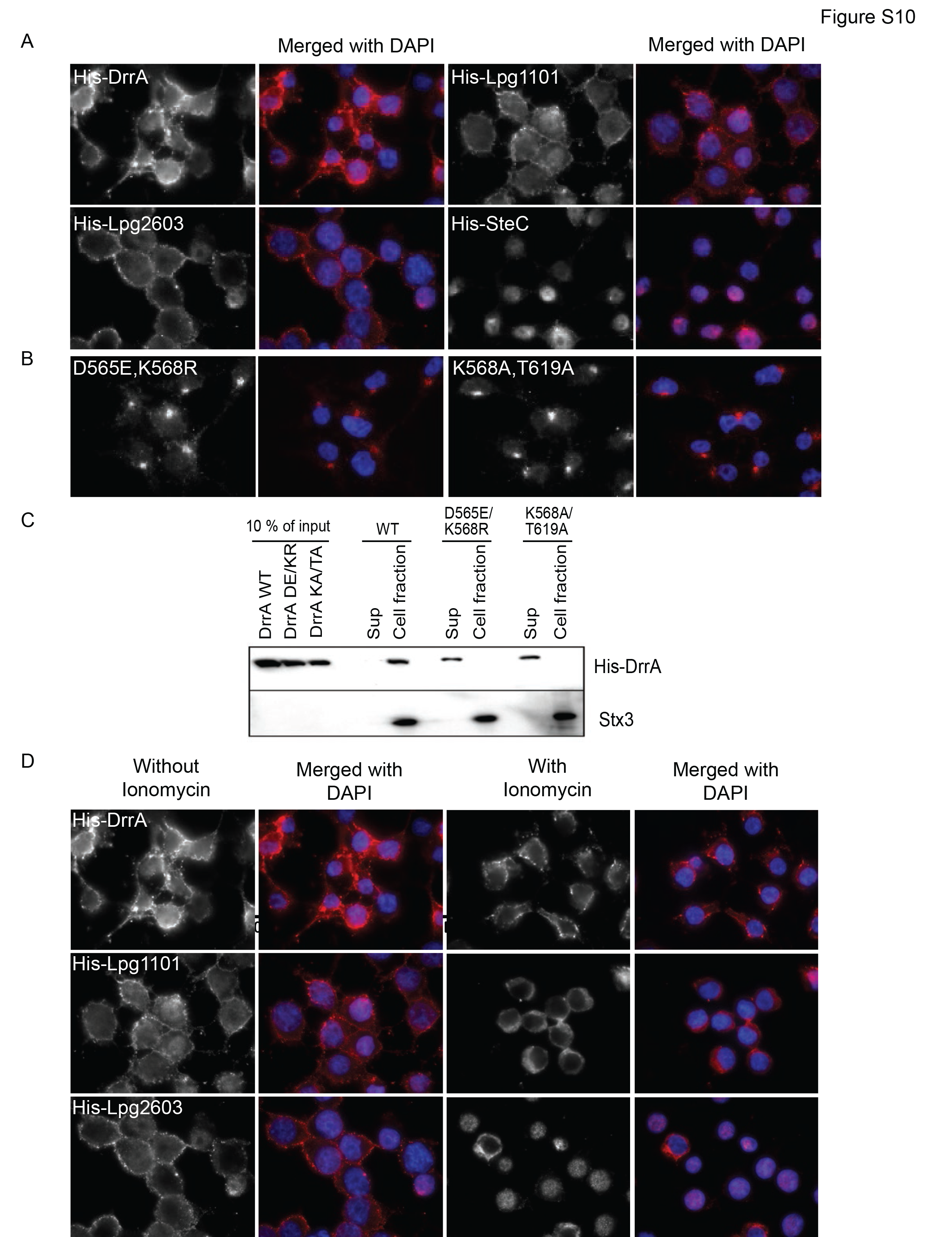

Supplement: Figure S10 — Semi-permeabilized cell system to examine LEPR domain localization. (A) Epifluorescent images of HEK293 FcγRII cells treated with digitonin and then incubated with purified His-tagged proteins DrrA, Lpg1101 and Lpg2603 from Legionella pneumophila or with a non-LEPR containing bacterial protein (His-SteC from Salmonella enterica serover Typhimurium). After washing away non-bound protein, proteins were detected using an anti-His antibody. (B) Micrographs showing localization of HisDrrA D565E/K568R, and HisDrrA K568A/T619A in semi-permeabilized cell system as in (A). Note that purified DrrA, with mutations that perturb PI4P-binding, show Golgi-localization. This is consistent with targeting due to Rab1-binding (see Figure S1C). (C) Fractionation of HEK293 cells after addition of His-DrrA, HisDrrA D565E/K568R, K568A/T619A in the semi-permeabilized cell system. The lower blot shows detection of Syntaxin 3 (Stx3) as a membrane fraction control. (D) Representative micrographs showing results of semi-permeabilized assay to assess the influence of ionomycin on localization of purified His-DrrA, His-Lpg1101, and His-Lpg2603. (TIF) [file ppat.1004222.s010.tif]

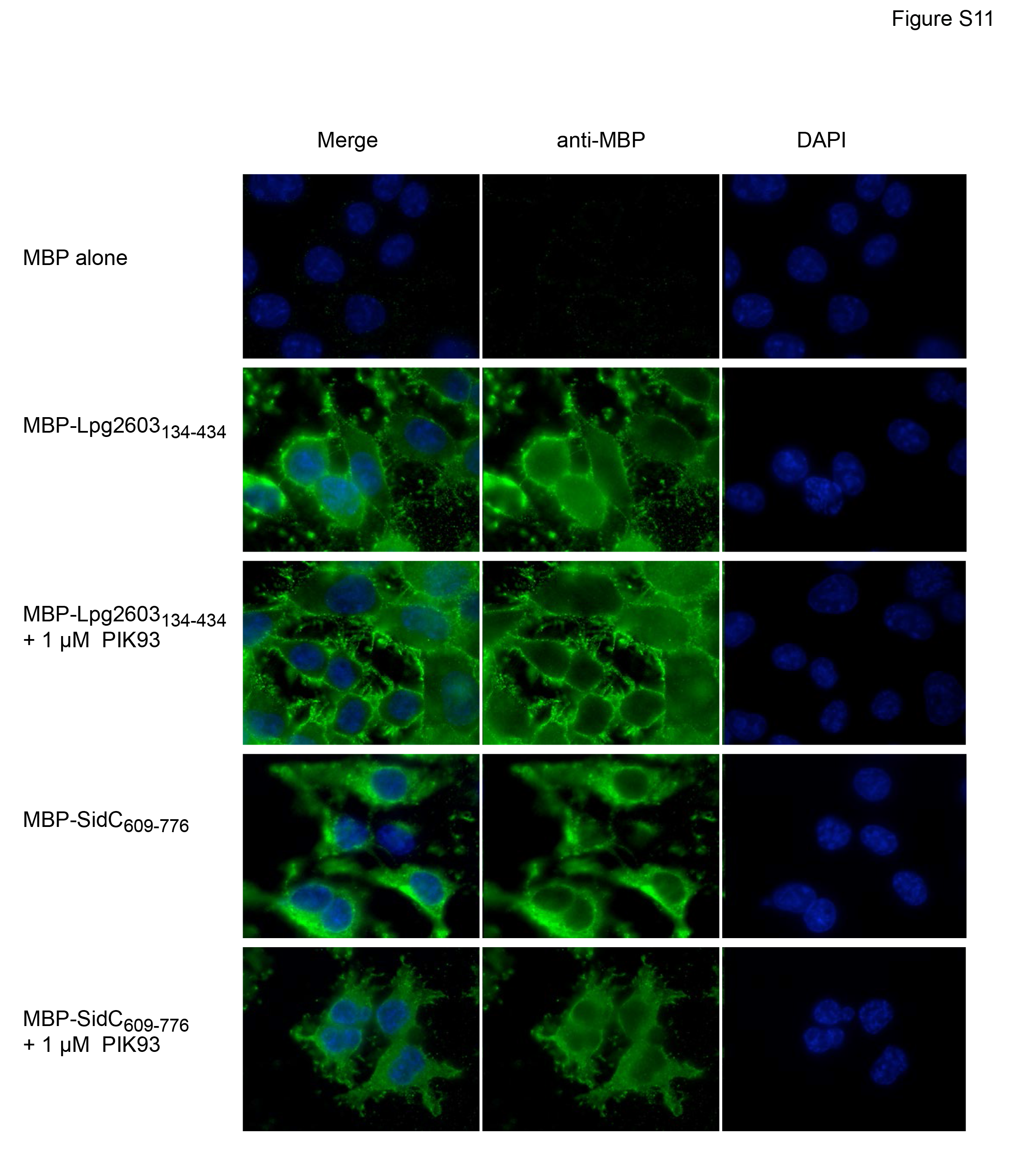

Supplement: Figure S11 — SidC localizes to the Golgi-apparatus in a PI4KIIIβ-dependent manner. Epifluorescent images of HEK293 FcγRII cells treated with digtonin and then incubated with purified MBP-Lpg2603134–434 or MBPSidC609–776. After washing away non-bound protein, proteins were detected using an anti-MBP antibody. When indicated cells were preincubated with 1 µM PIK93 for 30 min prior to digitonin treatment. PIK93 was also maintained throughout the protein binding step. PIK93 causes a change in the localization of the Golgi-localized PI4P-binding probe from SidC but not the PM-localized Lpg2603 probe. (TIF) [file ppat.1004222.s011.tif]

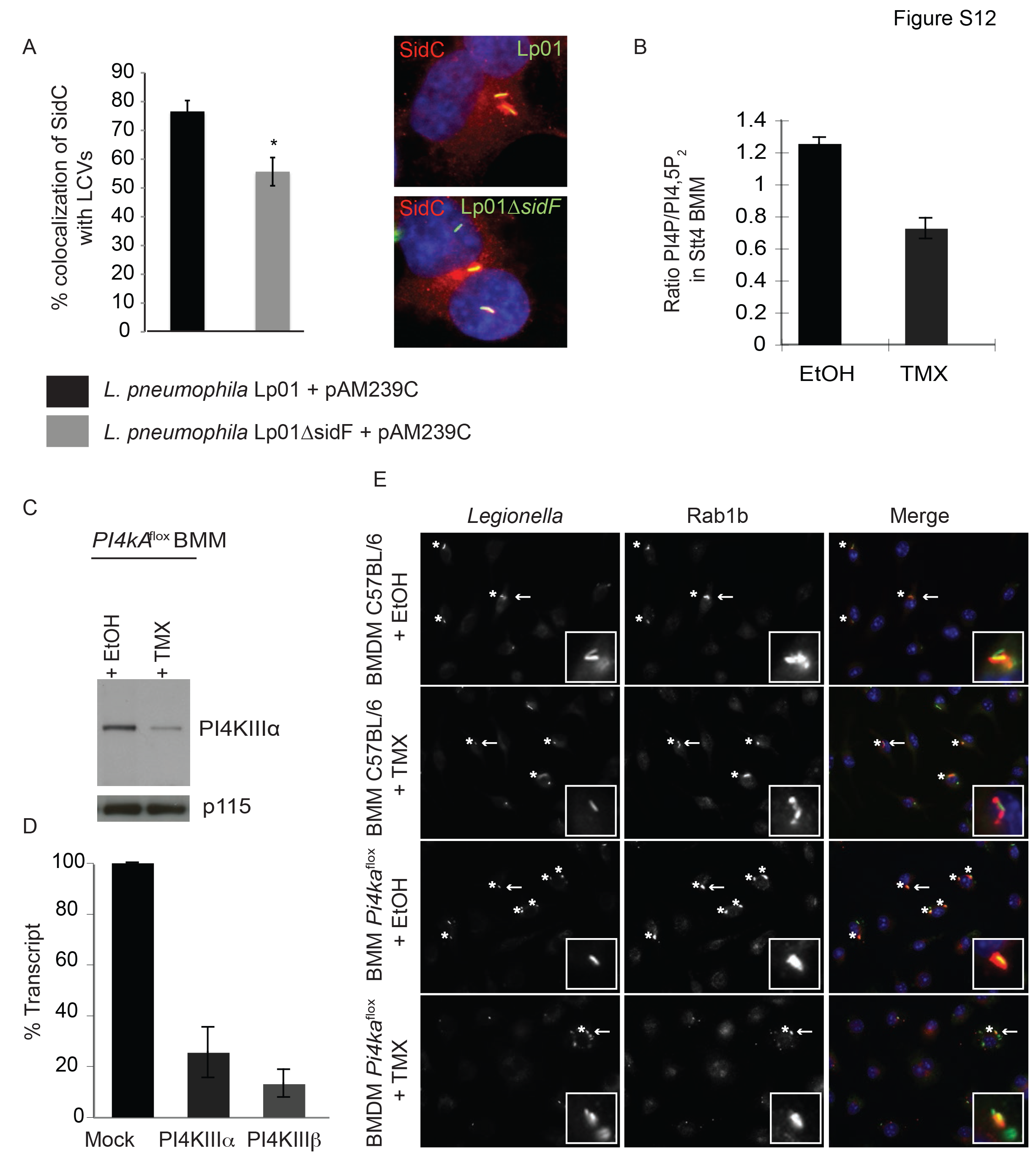

Supplement: Figure S12 — Controls for siRNA knockdown and cre-mediated deletion of PI4KIIIα in Pi4kaflox –derived cells. (A) Quantification of SidC-recruitment to vacuoles formed by wild type or ΔsidF mutant L. pneumophila expressing GFP (+pAM239C) in HEK FcγRII cells after 1 hour of infection. Fluorescent micrographs show representative staining pattern of SidC used for analysis. (B) Anion-exchange HPLC of glycerol-inositol phosphates obtained from control and knockout BMM. Data shown are the mean ratio of PI4P to PI(4,5)P2 levels (peak areas), which represent the bulk of PIP2 and PIP respectively, from three independent experiments. (C) Western-blot showing protein levels of PI4KIIIα at day 7 after TMX (tamoxifen) treatment or EtOH (ethanol vehicle control) treatment. Also shown is the level of the protein p115. (D) The extent of knockdown of PI4KIIIα and PI4KIIIβ mRNA by siRNA in HEK293 FcγRII cells was assessed by RT-PCR on cDNA obtained from mRNA extracted from cells on day 3. For each sample, transcript levels of genes targeted for silencing were compared to transcript levels of GAPDH. Results shown are the average of three independent experiments, and are presented as the percent of transcript detected relative to mock controls. (E) Representative micrographs taken with an epifluorescent microscope using a 60× objective showing BMDM infected with GFP expressing L. pneumophila. Asterisks indicated LCVs scored positive for Rab1 recruitment. (TIF) [file ppat.1004222.s012.tif]
