# Supplementary material for: The Machinery at Endoplasmic Reticulum-Plasma Membrane Contact Sites Contributes to Spatial Regulation of Multiple Legionella Effector Proteins
Source: PLoS Pathog. 2014 Jul 3;10(7):e1004222. doi: 10.1371/journal.ppat.1004222 (PMC4081824; doi:10.1371/journal.ppat.1004222)
Supplement: Text S1 — Supplementary information including Tables S1–S5, and additional methods and references for supporting figures. Tables S1–S3 list the bacterial strains, plasmids and primers used in this study, respectively. Table S4 provides the sequences of siRNAs and Table S5 lists primers used for RT-PCR. (DOCX) [file ppat.1004222.s013.docx]

**Supplementary Tables**

**Table S1. Bacterial strains**

| **Strain** | **Genotype (name in study)** | **Reference** |
| --- | --- | --- |
| *Legionella* |  |  |
| Lp01 (CR39) | *Legionella pneumophila* serogroup 1, Lp01 *rpsL* | [[1](#_ENREF_1)] |
| Lp01∆*dotA* (CR58) | Lp01∆*dotA rpsL* | [[2](#_ENREF_2)] |
| Lp01∆*drrA* | Lp01∆*drrA rpsL* | [[3](#_ENREF_3)] |
| Lp01 dsRed | Lp01 + pMMB207::dsRed | [[4](#_ENREF_4)] |
| Lp01∆*dotA* dsRed | Lp01 + pMMB207::dsRed | [[4](#_ENREF_4)] |
| Lp01 GFP | Lp01 + pAM239C | This study |
| Lp01∆*lpg1101* | Lp01∆*lpg1101 rpsL* | This study |
| Lp01∆*lpg2603* | Lp01∆*lpg2603 rpsL* | This study |
| Lp01∆*sidF* | Lp01∆*sidF rpsL* | This study |
| Lp01∆*sidF* pAM239C | Lp01∆*sidF* + pAM239C *rpsL* | This study |
| Lp01∆*drr* pJB*drrA* | Lp01∆*drrA rpsL* (pJB*drrA*) | This study |
| Lp01∆*drrA* pJB*drrA* D/E, K/R | Lp01∆*drrA rpsL* (pJB*drrA* D/E, K/R) | This study |
| Lp01∆*drrA* pJB*drrA* K/A, T/A | Lp01∆*drrA rpsL* (pJB*drrA* K/A, T/A) | This study |
| Lp01∆*drrA* pJB*lpg1101* | Lp01∆*drrA rpsL* (pJB*lpg1101*) | This study |
| Lp01∆*drrA* pJB*lpg2603* | Lp01∆*drrA rpsL* (pJB*lpg2603*) | This study |
| Lp01*∆lpg1101* pJB*lpg1101* | Lp01*∆lpg1101 rpsL* (pJB*lpg1101*) | This study |
| Lp01∆*lpg1101* pJB*lpg1101* K/A, T/A | Lp01∆*lpg1101 rpsL* (pJB*lpg1101* K/A, T/A) | This study |
| Lp01*∆lpg2603* pJB*lpg2603* | Lp01*∆lpg2603 rpsL* (pJB*lpg2603*) | This study |
| Lp01∆*lpg2603* pJB*lpg2603* K/A, T/A | Lp01∆*lpg2603 rpsL* (pJB*lpg2603* K/A, T/A) | This study |
| pCya | Lp01 *rpsL* + pJV450 M45Cya | [[5](#_ENREF_5)] |
| pCya::LepA | Lp01 *rpsL* + pJV450*lepA* | Gifted by A. Ingmundson |
| pCya::Lpg1101 | Lp01 *rpsL* + pJV450*lpg1101* | This study |
| ∆*dotA* pCya::Lpg1101 | Lp01∆*dotA* + pJV450*lpg1101* | This study |
| pCya::Lpg2603 | Lp01 *rpsL* + pJV450*lpg2603* | This study |
| *∆dotA* pCya::Lpg2603 | Lp01∆*dotA* + pJV450*lpg2603* | This study |
| Lp02 | Thymidine auxotroph derived from of strain Lp01 | [[1](#_ENREF_1)] |
| JR32∆*flaA* | JR32∆*flagellin* | [[6](#_ENREF_6)] |
| JR32∆*flaA* GFP | JR32∆*flagellin* + pAM239 | Gifted by Sunny Shin |
| *E.coli* |  |  |
| DH5α | endA1 hsdR17 supE44 thi-1 recA1 gyrA relA1 Δ(lacIZYA-argF)U169, deoR (φ80dlacΔ(lacZ)M15) | Invitrogen |
| CR014 | DH5α (λ pir) | [[2](#_ENREF_2)] |
| CR019 | MT607 *E. coli* containing plasmid pRK600; ColE1 replicon with RK2 transfer genes, Cm^R^ | [[7](#_ENREF_7)] |

**Table S2. Plasmids**

| **Short name** | **Plamids** | **Properties** | **Reference** |
| --- | --- | --- | --- |
|  | pJB1806 | RSF1010 ori, -tdΔi, Ap^R^, ΔoriT, Cm^R^ | [[8](#_ENREF_8)] |
| pAH1 | pJB1806M45 | pJB1806 modified to contain *icmR* promoter and an amino-terminal M45 epitope-tag | This study |
| pAH2 | pJB*drrA* | pJB1806 M45 encoding M45-*drrA* 1-647 | This study |
| pAH3 | pJB*drrA* D/E, K/R | pJB*drrA* with D/E and K/R substitutions at positions 565 and 568, respectively | This study |
| pAH4 | pJB*drrA* K/A, T/A | pJB*drrA* with K/A and T/A substitutions at positions 568 and 619, respectively | This study |
| pAH5 | pJB*lpg1101* | pJB1806 M45 encoding M45-*lpg1101* | This study |
| pAH6 | pJB*lpg1101* K/A, T/A | pJB*lpg1101* with K/A and T/A substitutions at positions 246 and 297, respectively | This study |
| pAH7 | pJB*lpg2603* | pJB1806 M45 encoding M45-*lpg2603* | This study |
| pAH8 | pJB*lpg2603* K/A, T/A | pJB*lpg1101* with K/A and T/A substitutions at positions 358 and 402, respectively | This study |
|  | pJV450 M45Cya | N-terminal M45Cya fusion vector, Km^R^ | [[5](#_ENREF_5)] |
|  | pJV450*lepA* | pJV450 M45Cya encoding *lepA* | Gift from A. Ingmundson |
| pAH9 | pJV450*lpg1101* | pJV450 M45Cya encoding *lpg1101* | This study |
| pAH10 | pJV450*lpg2603* | pJV459 M45Cya encoding *lpg2603* | This study |
|  | pSR47S | R6K suicide vector, Km^R^, sacB | [[9](#_ENREF_9)] |
| pAH11 | pSR*lpg1101* | pSR47S containing 5’ and 3’ flanking genomic regions of *lpg1101* (for gene deletion) | This study |
| pAH12 | pSR*lpg2603* | pSR47S containing 5’ and 3’ flanking genomic regions of *lpg2603* (for gene deletion) | This study |
|  | pET15b | Expression vector for Histidine-tagged proteins, Ap^R^ | Novagen |
|  | pH6-*drrA*_1-647_ | pET15b encoding his-tagged *drrA*_1-647_ | [[3](#_ENREF_3)] |
|  | pH6-*drrA*_1-500_ | pET15b encoding his-tagged *drrA*_1-500_ | [[3](#_ENREF_3)] |
| pAH13 | pET*lpg1101* | pET15b encoding his-tagged *lpg1101* | This study |
| pAH14 | pET*lpg2603* | pET15b encoding his-tagged *lpg2603* | This study |
|  | pMAL-c5X | Expression vector for MBP-tagged proteins, Ap^R^ | New England Biolabs Inc. |
| pAH15 | pMAL*fapp1* | pMAL-c5X encoding MBP-tagged FAPP1_1-100_ | This study |
| pAH16 | MBPSidC_609-776_ | pMAL-c5x encoding MBP-SidC_609-776_ | This study |
| pAH17 | pMAL*drrA_451-647_* | pMAL-c5x encoding MBP-tagged *drrA*_451-647_ | This study |
| pAH18 | pMAL*drrA_501-647_* | pMAL-c5x encoding MBP-tagged *drrA*_501-647_ | This study |
| pAH19 | pMAL*drrA_501-647_* L610A/614A/615A/617A | pMAL-c5x encoding MBP-tagged *drrA*_501-647_ L610A/614A/615A/617A | This study |
| pAH20 | pMAL*lpg1101* | pMAL-c5x encoding MBP-tagged *lpg1101* | This study |
| pAH21 | pMAL*lpg1101* L288A/I295A | pMAL-c5x encoding MBP-tagged *lpg1101* L288A/I295A mutant | This study |
| pAH22 | pMAL*lpg1101* V292A/T293A/I295A | pMAL-c5x encoding MBP-tagged *lpg1101* V292A/T293A/I295A | This study |
| pAH23 | pMAL*lpg1101* L288A/V292A/T293A/I295A | pMAL-c5x encoding MBP-tagged *lpg1101* L288A/V292A/T293A/I295A | This study |
| pAH24 | pMAL*lpg1101_180-323_* | pMAL-c5x encoding MBP-tagged *lpg1101*_180-323_ | This study |
| pAH25 | pMAL*lpg2603* | pMAL-c5x encoding MBP-tagged *lpg2603* | This study |
| pAH26 | pMAL*lpg2603* L405A | pMAL-c5x encoding MBP-tagged *lpg2603* L405A mutant | This study |
| pAH27 | pMAL*lpg2603_289-435_* | pMAL-c5x encoding MBP-tagged *lpg2603*_289-435_ | This study |
|  | pGFP-*drrA*_61-647_ | pEGFP encoding *gfp-drrA*_61-647_ | [[3](#_ENREF_3)] |
|  | dsRED | pMMB207 containing RED | [[10](#_ENREF_10)] |
|  | pAM239 | pMMB207 containing gfpmut3 | [[10](#_ENREF_10)] |
|  | pAM239C | pAM239 digested with Mlu1 enzyme and relegated resulting in internal deletion within the lacIQ | This study, provided by Hiroki Nagai |
| pAH28 | pGFP-*drrA*_61-647_ K/A | *gfp-drrA*_61-647_ with K/A substitution at positions 568 | This study |
| pAH29 | pGFP-*drrA_61-647_* D/E, K/R | *gfp-drrA*_61-647_ with D/E and K/R substitutions at positions 565 and 568, respectively | This study |
| pAH30 | pGFP-*drrA_61-647_* K/A, T/A | *gfp-drrA*_61-647_ with K/A and T/A substitutions at positions 568 and 619, respectively | This study |
| pAH31 | pGFP-*drrA_501-647_* | *gfp-drrA*_501-END_ | This study |
| pAH32 | pGFP-*drrA_501-647_* L610A | pAH31 with L610A mutation | This study |
| pAH33 | pGFP-*drrA_501-647_* L617A | *gfp-drrA*_501-END_ with L617A mutation | This study |
| pAH34 | pGFP-*drrA_501-647_* L614A/615A | pAH31 with L614A/615A mutation | This study |
| pAH35 | pGFP-*drrA_501-647_* L610A/614A/615A | pAH34 with additional L610A mutation, created with primers 41/42 | This study |
| pAH36 | pGFP-*drrA_501-647_* L614A/615A/617A | pAH34 with additional L617A mutation, created with primers 43/44 | This study |
| pAH37 | pGFP-*drrA_501-647_* L610A/L614A/615A/617A | pAH36 with additional L610A mutations, created with primers 41/42 | This study |
|  | pEYFP-C1 | EYFP expression vector, Km^R^ | Clontech |
| pAH38 | pYFP-*drrA*_451-647_ | pEYFP-C1 encoding e*yfp-drrA*_451-647_ | This study |
| pAH39 | pYFP-*lpg1101* | pEYFP-C1 encoding e*yfp-lpg1101* | This study |
| pAH40 | pYFP-*lpg2603* | pEYFP-C1 encoding e*yfp-lpg2603* | This study |
| pAH41 | pYFP-*lpg1101*_180-323_ | pEYFP-C1 encoding e*yfp-lpg1101_180-323_* | This study |
| pAH42 | pYFP-*lpg2603*_289-435_ | pEYFP-C1 encoding e*yfp-lpg2603_289-435_* | This study |
| pAH43 | pYFP-*lpg1101_225-323_* | pEYFP-C1 encoding e*yfp*-*lpg1101_225-323_* | This study |
| pAH44 | pYFP-*lpg2603*_334-435_ | pEYFP-C1 encoding e*yfp-lpg2603_334-435_* | This study |
| pAH45 | pYFP-*lpg2603_1-316_* | pEYFP-C1 encoding *yfp-2603_1-316_* | This study |
| pAH46 | pYFP-*lpg1101* D/E, K/R | pAH39 with D/E and K/R substitutions at positions 243 and 246, respectively | This study |
| pAH47 | pYFP-*lpg1101* K/A, T/A | pAH39 with K/A and T/A substitutions at positions 246 and 297, respectively | This study |
| pAH48 | pYFP-*lpg1101* V292A | pAH39 with V292A mutation | This study |
| pAH49 | pYFP-*lpg1101* I295A | pAH39 with I295A mutation | This study |
| pAH50 | pYFP-*lpg1101* V292A/I295A | pAH39 with V292A/I295A mutations | This study |
| pAH51 | pYFP-*lpg1101* V292A/T293A | pAH39 with V292A/T293A mutations | This study |
| pAH52 | pYFP-*lpg1101* V292A/T293A/I295A | pAH49 with additional V292A/T293A mutations | This study |
| pAH53 | pYFP-*lpg1101* L288A/V292A/T293A/I295A | pAH52 with additional L288A mutation | This study |
| pAH55 | pYFP-*lpg2603* D/E, K/R | pAH40 with D/E and K/R substitutions at positions 355 and 358, respectively | This study |
| pAH56 | pYFP-*lpg2603* K/A, T/A | pAH40 with K/A and T/A substitutions at positions 358 and 409, respectively | This study |
| pAH57 | pYFP-*lpg2603* D/E | pAH40 with D/E substitutions at position 355 | This study |
| pAH58 | pYFP-*lpg2603* K/R | pAH40 with K/R substitutions at position 358 | This study |
| pAH59 | pYFP-*lpg2603* G/A | pAH40 with G/A substitutions at position 354 | This study |
| pAH60 | pYFP-*lpg2603* L/A | pAH40 with L/A substitutions at position 357 | This study |
| pAH61 | pYFP-*lpg2603* K/A | pAH40 with K/A substitutions at position 358 | This study |
| pAH62 | pYFP-*lpg2603* I/A | pAH40 with I/A substitutions at position 361 | This study |
| pAH63 | pYFP-*lpg2603* T/A | pAH40 with T/A substitutions at position 409 | This study |
| pAH64 | pYFP-*lpg2603* L405A | pAH40 with L/A substitutions at position 405 | This study |
| pAH65 | pSR*sidF* | pSR47S containing 5’ and 3’ flanking genomic regions of *sidF* (for gene deletion) | This study |
| pAH66 | pYES2*lpg2603* | pYES2-*lpg2603* | This study |
| pAH67 | pYES2*lpg2603_G354A_* | pAH66 with G354A substitution | This study |
| pAH68 | pYES2*lpg2603_D355A_* | pAH66 with D355A substitution | This study |
|  | mtagRFP-PALM |  | Gift from Brett Lindenbach |

**Table S3. Primers used for cloning**

| **Primer number** | **Primer name** | **Sequence** | **Construct** |
| --- | --- | --- | --- |
| 1 | icmR-M45_5’ | tgat**GAATTC**ATAGTTGTTTGTAAAGAATTAGAA | pJB1806 M45 |
| 2 | icmR-M45 _3’ | gcct**GGATCC**GCGTCTCTGTCTCA | pJB1806M45 |
| 3 | pJBDrrA_5’ | CCC**GGATCC**TTATGAGCATAATGGG | pJB*drrA* |
| 4 | pJBDrrA_3’ | GTTCAA**GTCGAC**TTATTTTATCTTAAT | pJB*drrA* |
| 5 | pJB1101_5’ | catt**ggatcc**taATGACTACGTCGGTGTCTTTT | pJB*lpg1101* |
| 6 | lpg1101_Sal1_3’ | agaa**gtcgac**CTACCTCATTGCAAGCTTGG | pJB*lpg1101,*  pEYFP*lpg1101* |
| 7 | pJB2603_5’ | catt**ggatcc**taTTGTATTCTGATTTTTTTTTCAC | pJB*lpg2603* |
| 8 | lpg2603_Sal1_3’ | agaa**gtcgac**CTAAATGCTTACATTAGGGCT | pJB*lpg2603,* pEYFP*lpg2603* |
| 9 | lpg1101_up_5’ | cg**gagctc**TGCCCTATTATTGATGTTACCAAT | pSR*lpg1101* |
| 10 | lpg1101_up_3’ | CAAATTTGAGCATGATTATTTCCTCGCAAAACC | pSR*lpg1101* |
| 11 | lpg1101_down_5’ | AATAATCATGCTCAAATTTGATTGGGCAGTTG | pSR*lpg1101* |
| 12 | lpg1101_down_3’ | gc**tctaga**AACGATAGCCAAGAGTGGTAA | pSR*lpg1101* |
| 13 | lpg2603_up_5’ | gc**gagctc**TATGATCCAATGCAATCTGGT | pSR*lpg2603* |
| 14 | lpg2603_up_3’ | TGGACACGTCCAAATGAGATGGTTGATGAGT | pSR*lpg2603* |
| 15 | lpg2603_down_5’ | ATCTCATTTGGACGTGTCCATTTTAAATTCTG | pSR*lpg2603* |
| 16 | lpg2603_down_3’ | CG**TCTAGA**TTCTCCATCTTCATAAAGACC | pSR*lpg2603* |
| 17 | lpg1101-pET_1 5’ | cgc**ggatcc**CATGACTACGTCGGTGTCTTTT | pET*lpg1101* |
| 18 | lpg1101-pET_3’ | taa**ggatcc**TGGCAACTGCCCAATCAAATTTGAG | pET*lpg1101* |
| 19 | lpg2603-pET_1 5’ | cgc**ggatcc**TTTGTATTCTGATTTTTTTTTCACTATAATG | pET*lpg2603* |
| 20 | lpg2603-pET_3’ | tca**ggatc**cTCACAGAATTTAAAATGGACACGTC | pET*lpg2603* |
| 21 | FAPP1PH_5’ | AGATTC**AGATCT**atggagggggtgttgtacaag | pMAL*fapp1* |
| 22 | FAPP1PH_3’ | TTCGGT**gaattc**TCAagtccttgtatcagtcaaac | pMAL*fapp1* |
| 23 | FAPP1R18L_f | cacaggctggcagcctctttggtttgttttagataa | pMAL*fapp1* R18L |
| 24 | FAPP1R18L_r | ttatctaaaacaaaccaaagaggctgccagcctgtg | pMAL*fapp1* R18L |
| 25 | DrrA_451__5’ | ggttta**GGATCC**attcaaaaacttgagagaag | pMAL*drrA*_451-647_, pEYFP*drrA*_451-647_ |
| 26 | DrrA_3’ | ttagtt**GAATTC**ttattttatcttaatggttt | pMAL*drrA*_451-647_, pEYFP*drrA*_451-647_ |
| 27 | DrrA_501__5’ | ATTG**CATATG**attcaaaaacttgagagaag | pMAL*drrA*_501-647_ |
| 28 | DrrA_501__3’ | ctaa**GGATCC**ttattttatcttaatggttt | pMAL*drrA*_501-647_ |
| 29 | DrrA D565E/K568R_f | ctggctgatttcaaggataaactggctgaa | Site mutants in *drrA* cloned into pJB1806, pET, pMAL-c5x or pEGFP |
| 30 | DrrA D565E/K568R_r | gatttctgttcttaaagcctcacctcgcat |  |
| 31 | DrrAK568A_f | tcagcaaatgcgaggtgatgctttagcaacagaaatcctggct |  |
| 32 | DrrAK568A_r | agccaggatttctgttgctaaagcatcacctcgcatttgctga |  |
| 33 | DrrAT619A_f | cccagcttttagggttaaaggcaagttcagtgtcttcattt |  |
| 34 | DrrAT619A_r | aaatgaagacactgaacttgcctttaaccctaaaagctggg |  |
| 35 | DrrA L610A_f | ttggctaaggggcaaggggcaacaacccagcttttagg |  |
| 36 | DrrA L610A_r | cctaaaagctgggttgttgccccttgccccttagccaa |  |
| 37 | DrrA L617A_f | gttaacaacccagcttttaggggcaaagacaagttcagtgtcttca |  |
| 38 | DrrA L617A_r | tgaagacactgaacttgtctttgcccctaaaagctgggttgttaac |  |
| 39 | DrrA L614A/615A_f | ggggcaagggttaacaacccaggctgcagggttaaagacaagttcagtg |  |
| 40 | DrrA L614A/615A_r | cactgaacttgtctttaaccctgcagcctgggttgttaacccttgcccc |  |
| 41 | DrrA L610A/614A/615A_f | gctaaggggcaaggggcaacaacccaggctgc |  |
| 42 | DrrA L610A/614A/615A_r | gcagcctgggttgttgccccttgccccttagc |  |
| 43 | DrrA L614A/615A/617A_f | acaacccaggctgcaggggcaaagacaagttcagtgtc |  |
| 44 | DrrA L614A/615A/617A_r | gacactgaacttgtctttgcccctgcagcctgggttgt |  |
| 45 | lpg1101-C1_5’ | ttt**agatct**ATGACTACGTCGGTGTCTTT | pYFP *1101** |
| 46 | lpg1101_180__5’ | GAGCAT**GGATCC**ttccataacgaaaagccagg | pYFP*1101*_180-323_, pMAL*1101*_180-323_* |
| 47 | lpg1101_225__5’ | GAGCAT**GGATCC**gatgaaaaaacaggtgaccaatc | pYFP*1101*_225-323_, pMAL*1101*_225-323_* |
| 48 | lpg1101_3’ | ttagtt**GAATTC**ctacctcattgcaagcttgg | pYFP*1101*_180-323_, pMAL*1101*_180-323_, pYFP*1101*_225-323_, pMAL*1101*_225-323_* |
| 49 | lpg1101 D243E, K246R_f | tagatacagtcaagtaaaaggagagatagtaagacgcgcaataattaataatttaa | Site mutants in *lpg1101* cloned in pJB1806, pET, pMAL-c5x or pEYFP |
| 50 | lpg1101 D243E, K246R_r | ttaaattattaattattgcgcgtcttactatctctccttttacttgactgtatcta |  |
| 51 | lpg1101 K246A_f | gatagatacagtcaagtaaaaggagatatattagcacgcgcaataattaataatttaa |  |
| 52 | lpg1101 K246A_r | ttaaattattaattattgcgcgtgctaatatatctccttttacttgactgtatctatc |  |
| 53 | lpg1101 L288A_f | caaagcccaagggttatttactaaagctgctggcataaaaactgattcg |  |
| 54 | lpg1101 L288A_r | cgaatcagtttttatgccagcagctttagtaaataacccttgggctttg |  |
| 55 | lpg1101 V292A_f | gcccaagggttatttactaaagctactggcataaaaactgattcg |  |
| 56 | lpg1101 V292A_r | cgaatcagtttttatgccagtagctttagtaaataacccttgggc |  |
| 57 | lpg1101 I295A_f | ggttatttactaaagttactggcgcaaaaactgattcgcaacgggcag |  |
| 58 | lpg1101 I295A_r | ctgcccgttgcgaatcagtttttgcgccagtaactttagtaaataacc |  |
| 59 | lpg1101 V292A/I295A_f | cccaagggttatttactaaagctactggcgcaaaaactgattcgcaacggg |  |
| 60 | lpg1101 V292A/I295A_r | cccgttgcgaatcagtttttgcgccagtagctttagtaaataacccttggg |  |
| 61 | lpg1101 V292A/T293A_f | ttatttactaaagctgctggcgcaaaaactgattcgcaacgggc |  |
| 62 | lpg1101 V292A/T293A_r | gcccgttgcgaatcagtttttgcgccagcagctttagtaaataa |  |
| 63 | lpg1101 T297A_f | gttatttactaaagttactggcataaaagctgattcgcaacggg |  |
| 64 | lpg1101 T297A_r | cccgttgcgaatcagcttttatgccagtaactttagtaaataac |  |
| 65 | Cyalpg1101_5’ | at**ggcgcgcc**aATGACTACGTCGGTGTCTTT | pJV450Cya-*lpg1101* |
| 66 | Cyalpg1101_3’ | at**ggcgcgcc**aAACTGCCCAATCAAATTTGAGCTA | pJV450Cya-*lpg1101* |
| 67 | lpg2603-C1_5’ | ttt**agatct**TTGTATTCTGATTTTTTTTTCAC | pYFP*lpg2603*  pYES2*lpg2603* |
| 68 | lpg2603_289__5’ | GAGCAT**GGATCC**tcggataatccccatacgtt | pYFP*2603*_289-435_, pMAL*2603*_289-435_* |
| 69 | lpg2603_334__5’ | GAGCAT**GGATCC**gaggtaggggtgaatcgctt | pYFP*2603*_334-435_, pMAL*2603*_334-435_* |
| 70 | lpg2603_3’ | ttagtt**GAATTC**ctaaatgcttacattagggc | pYFP*2603*_289-435_, pMAL*2603*_289-435_, pYFP*2603*_334-435_, pMAL*2603*_334-435_* |
| 71 | lpg2603 D355E, K358R_f | aatctatgagggtttaaagggtgaggggctacgaaaagtgatcctgaaagaacttag | Site mutants in *lpg2603* cloned in pJB1806, pET, pMAL-c5x or pEYFP |
| 72 | lpg2603 D355E, K358R_r | ctaagttctttcaggatcacttttcgtagcccctcaccctttaaaccctcatagatt |  |
| 73 | lpg2603 G354A_f | gaaatctatgagggtttaaaggctgatgggctaaaaaaagtgat |  |
| 74 | lpg2603 G354A_r | atcactttttttagcccatcagcctttaaaccctcatagatttc |  |
| 75 | lpg2603 D355A_f | ctatgagggtttaaagggtgctgggctaaaaaaagtgatc |  |
| 76 | lpg2603 D355A_r | gatcactttttttagcccagcaccctttaaaccctcatag |  |
| 77 | lpg2603 D355E_f | gaaatctatgagggtttaaagggtgaggggctaaaaaaagtg |  |
| 78 | lpg2603 D355E_r | cactttttttagcccctcaccctttaaaccctcatagatttc |  |
| 79 | lpg2603 K359R_f | gggtttaaagggtgatgggctacgaaaagtgatcctgaaagaactt |  |
| 80 | lpg2603 K359R_r | aagttctttcaggatcacttttcgtagcccatcaccctttaaaccc |  |
| 81 | lpg2603 K358A_f | gggtttaaagggtgatgggctagcaaaagtgatcctgaaagaactt |  |
| 82 | lpg2603 K358A_r | gggtttaaagggtgatgggctagcaaaagtgatcctgaaagaactt |  |
| 83 | lpg2603 L357A_f | gagggtttaaagggtgatggggcaaaaaaagtgatcctgaaaga |  |
| 84 | lpg2603 L357A_r | tctttcaggatcactttttttgccccatcaccctttaaaccctc |  |
| 85 | lpg2603 I361A_f | gggtgatgggctaaaaaaagtggccctgaaagaacttagagattcg |  |
| 86 | lpg2603 I361A_r | cgaatctctaagttctttcagggccactttttttagcccatcaccc |  |
| 87 | lpg2603 L405A_f | gtcaggataagacaacaaaagcagcgaacttaaaaaccagttctcgta |  |
| 88 | lpg2603 L405A_r | tacgagaactggtttttaagttcgctgcttttgttgtcttatcctgac |  |
| 89 | lpg2603 T409A_f | ggataagacaacaaaagcactgaacttaaaagccagttctcgtaaaaa |  |
| 90 | lpg2603 T409A_r | tttttacgagaactggcttttaagttcagtgcttttgttgtcttatcc |  |
| 91 | lpg2603 R412G, K413A_f | aaagcactgaacttaaaaaccagttctggtgcaaaggtgatggtaatatttaaagaagc |  |
| 92 | lpg2603 R412G, K413A_r | gcttctttaaatattaccatcacctttgcaccagaactggtttttaagttcagtgcttt |  |
| 93 | lpg2603 F419A_f | ttctcgtaaaaaggtgatggtaatagctaaagaagcagaagagcgaatact |  |
| 94 | lpg2603 F419A_r | agtattcgctcttctgcttctttagctattaccatcacctttttacgagaa |  |
| 95 | Cyalpg2603_5’ | tt**ggcgcgcc**aTTGTATTCTGATTTTTTTTTCACTATAATG | Cya*lpg2603* |
| 96 | Cyalpg2603_3’ | at**ggcgcgcc**aAGAATTTAAAATGGACACGTC | Cya*lpg2603* |
| 97 | sidF down 5’ | cg**gagctc**TGTCTCAAGTTCGCCTCAA | pSR*sidF* |
| 98 | sidF down 3’ | CTCATAACCCCATGGTTTATTCCATATCATAAC |  |
| 99 | sidF up 5’ | ATAAACCATGGGGTTATGAGTTTAACAATCTA |  |
| 100 | sidF up 3’ | gc**tctaga**GAGCAATATAACCACGGTCA |  |
| 101 | sidC 607-776 5’ | gt**catatg**AAATATTCCTCCAAGCCATT | pMALc5xSidC 607-776 |
| 102 | sidC 607-776 3’ | ca**ggatcc**tcaAAAGAATTCAATTGCTTCA |  |
| 103 | lpg2603 Sal 3’ | agaa**gtcgac**CTAAATGCTTACATTAGGGCT | pYES2*lpg2603* (cloned into Xho1) |
| 104 | lpg2603 D355A _f | ctatgagggtttaaagggtgctgggctaaaaaaagtgatc | pYES2 2603_D355A_* |
| 105 | lpg2603 D355A _r | gatcactttttttagcccagcaccctttaaaccctcatag | pYES2 2603_D355A*_ |
| 106 | lpg2603 G354A _f | gaaatctatgagggtttaaaggctgatgggctaaaaaaagtgat | pYES2 2603_G354A_* |
| 107 | lpg2603 G354A _r | atcactttttttagcccatcagcctttaaaccctcatagatttc | pYES2 2603_G354A_* |

*Note: for brevity *lpg1101* and *lpg2603* are abbreviated to 1101 and 2603.

**Table S4. Sequence of individual siRNAs used in this study.**

| Name | Target sequence |
| --- | --- |
| Human PI4KA A | GUGAAGCGAUGUGGAGUUA |
| Human PI4KA B | CCACAGGCCUCUCCUACUU |
| Human PI4KA C | GCAGAAAUUGGCCUGUUU |
| Human PI4KA D | CCAACAUGACUGAGCGCGA |
| Human PI4KB A | GGGAUGACCUUCGGCAAGA |
| Human PI4KB B | GAGAUCCGUUGCCUAGAUG |
| Human PI4KB C | GCACCGAGAGUAUUGAUAA |
| Human PI4KB D | GCUGAUUGCCGCUCGGAAA |

**Table S5. Primers used for qRT-PCR**

| Primer name | sequence |
| --- | --- |
| GAPDH_FW | \| acagtcagccgcatcttctt \| \| --- \| |
| GAPDH_RV | acgaccaaatccgttgactc |
| PI4K3A1_FW | \| tttggaccgccatgttct \| \| --- \| |
| PI4K3A1_RV | \| tccgggtgtcctgattatct \| \| --- \| |
| PI4K3A2_FW | \| aaggccagctccgttgtat \| \| --- \| |
| PI4K3A2_RV | tcactgccagggagcaat |
| PI4K3B_FW | tgcgtggaaattgaatgagat |
| PI4K3B_RV | gggcaggctccactactgt |

**Materials and Methods**

**Effector translocation assay**

Protein translocation of candidate substrates from *Legionella pneumophila* into host cells was assessed using an adenylate cyclase-based assay (Cya) as described previously [[11](#_ENREF_11)].

**Antibody dilutions**

Antibodies used for immunofluorescence were as follows anti-HA (1:300), anti-MBP (1:500), anti-His (1:500), anti-GM130 (1:50), anti-Lpg1101 (1:100), anti-Lpg2603 (1:100), anti-DrrA (1:300) anti-Rab1b (1:300). The rabbit anti-SidC polyclonal antibody, provided by Zhao-Qing Luo, was used at 1:100 dilution as previously described [[12](#_ENREF_12)]. Primary antibodies used for Western-blot analysis were as follows anti-p115 (1:50), anti-calnexin (1:1000), anti-tubulin (1:5000). Secondary horseradish peroxidase conjugates were used at 1: 3,000 and secondary Alexa Fluor^®^ dyes at 1:500-1:2000.

**GST-pull down assay**

Assays to assess binding of effectors to Rab1 were based on described methods [[13](#_ENREF_13)]. His-tagged effector proteins from post-nuclear supernatants (100 μg) were added to purified GST–Rab proteins (approximately 100 µg) immobilized on glutathione sepharose 4B (GE Healthcare). Binding reactions were performed in 2.5 ml of lysis buffer containing 0.2 mM GDP and 5 mM MgCl_2._ for 30 min at 4 °C. Washing of unbound protein was done with seven 1 ml washes as follows: three times with PBS with 1 mM DTT and 1% Triton X-100, 2 times with PBS with 1mM DTT, 1% Triton X-100 and 0.2 M NaCl, and finally twice with PBS with 1 mM DTT. Bound protein was eluted with 100 µl of 10 mM glutathione in 50 mM Tris-HCl pH 9.5 and 10% loaded onto SDS-page gels for Coomassie brilliant blue staining.

***L. pneumophila* growth assays.**

To analyze *L. pneumophila* growth in THP-1 cells, cells were seeded into white opaque 96-well tissue culture plates (BD-Falcon) at 1 x 10^5^/well along with 100 ng/ml PMA. After 24-30 hours the media was replaced without the addition of PMA. Two days later the media was replaced again and cells infected with *lux*-expressing *L. pneumophila* at MOI of 1 by adding 10 µl/well of 1 x 10 ^7^/ml bacterial solutions made by resuspending *Legionella* grown for 48 hours on solid growth media. All samples were represented by 8-12 wells per plate and averaged to give the final values for each experiment. Luminescence values were obtained using a PowerscanHT plate reader (DS Pharma Biomedical Ltd.) with the integration time set for 1 second/well with the default sensitivity setting. Luminescence growth assays were performed in triplicate. Standard colony forming unit (CFU) growth assays of *L. pneumophila* strains were performed in *A. castellanii* (ATCC strain 30234) and bone marrow derived macrophages from A/J mice as previously described [[11](#_ENREF_11)].

**3 x Flag co-immunoprecipitation**

HEK293 FcγRII cells were co-transfected with 3x-FLAG-Stx2, 3 or 4 and GFP-DrrA 200-500, 451-647, 501-647, 451-545 or 546-647 and with 3x-FLAG-Stx2, 3 or 4 and YFP-Lpg1101 or YFP-Lpg2603 using Lipofectamine™ 2000 (Invitrogen) according to manufacture's protocol with 1µg of plasmid DNA per well (6-well plate). At 20 hours post-transfection, cell lysates were prepared and immunoprecipitated by anti-FLAG M2 beads. Precipitated proteins were eluted, separated by SDS-PAGE and analyzed by antibodies against GFP and FLAG epitope. We also analyzed 2.5% or 4% of total lysates.

For preparing cell lysates, transfected cells were washed by PBS and lysed by lysis buffer containing 150mM KCl, 20mM Hepes-KOH (pH7.2), 2mM EDTA, 1% Triton X-100 and protease inhibitor cocktail. At 20 minutes after lysis, cells were centrifuged at *15,000 x g* to remove cell debris and resulting supernatant was used as lysate. Cell lysates were incubated with 5µl of FLAG-M2 antibody-conjugated agarose beads and incubated for 1 hour at 4˚C. After incubation, beads were washed with wash buffer containing 150mM KCl, 20mM Hepes-KOH (pH7.2), 2mM EDTA and 1% Triton X-100 three times. Precipitated proteins were eluted using buffer containing 150mM KCl, 20mM Hepes-KOH (pH 7.2), 2mM EDTA and 100µg/ml 3x-FLAG peptide.

A similar method for FLAG immunoprecipitation was used to confirm 3XFLAG-Lpg1101 expression in HEK293 cells stably transformed with this construct, except that lysis buffers contained 2 mM MgCl_2_ and EDTA was not included.

**PI4P probe localization assays**

Fluorescently-tagged probes containing PH-domains from CERT [[14](#_ENREF_14)], full-length FAPP1 (gifted by Isabelle Derre, Yale University), GFP-FAPP1R18L, and full-length GFPOCRL1 (accession number NM_000276.3) were transfected into HEK293 FcγRII cells. Primers for cloning of GFP-FAPP1 and for site-directed mutagenesis to create GFP-FAPP1R18L are listed in the primer table. After 24 hours, cells were infected using the standard protocol described earlier with dsRed expressing (pMMB207dsRed) wild type Lp01 or Lp01∆*dotA* (induced with 0.5 mM IPTG for 48 h prior to infection)*.* Cells were either fixed directly in 4% PFA and stained with DAPI before mounting or prepermeabilized to clear cytosolic proteins. To semi-permeabilize cells coverslips were first washed with PBS containing 0.9 mM CaCl_2_ and 1 mM MgCl_2_ and then incubated for 3 minutes with 0.1% saponin in PIPES buffer (80 mM PIPES, 5 mM EGTA, 1 mM MgCl_2_, pH 6.8) before fixing with 4% PFA. We were unable to detect a vacuolar localization signal for GFPOCRL1 or GFP-CERT and images are not shown.

**Quantitative RT-PCR analysis of siRNA knockdowns**

For real-time PCR analysis, total RNA was extracted from cells using the RNeasy® kit (Qiagen) with the addition of the option on-column DNase treatment using the RNase-Free DNase Set (Qiagen). First strand cDNA synthesis was performed on 1 µg of total RNA using SuperScript® II Reverse Transcriptase (RT) (Invitrogen) and the random Oligo(dT) primer as recommended by the manufacturer. In all first-strand synthesis reactions negative controls were included that lacked reverse transcriptase. Finally, the mRNA levels were determined by quantitative real-time PCR on an iCycler iQ system using iQ™ SYBR Green Supermix and the specific primers listed in Supplementary Table S4.

**Live Cell Imaging**

For live cell imaging experiments YFP-Rab1 expressing HEK293 FcγRII cells were seeded for 24-48 hours on 35mm poly-L-lysine-coated MatTek glass-bottom imaging chambers (MatTek Corporation, Ashland, MA). Before infection, media was replaced with phenol red-free RPMI (Gibco). *Legionella* strains were incubated with CellTracker™ Orange CMTMR (Invitrogen) for twenty minutes as described previously [[15](#_ENREF_15)]. To activate fluorescence, bacteria were resuspended in serum-free media for 30 minutes as per the manufacturer’s instructions. Fluorescent bacteria were resuspended in PBS and opsonized with rabbit anti-*Legionella* antibody at 1:1000. Antibody-bacteria complexes were added to cells at an MOI of 3. YFP-Rab1 HEK293 FcγRII cells and bacterial complexes were centrifuged for 1000rpm for 5 mins to facilitate synchronous uptake.

Imaging chambers containing infected cells were placed on a motorized XY stage enclosed by a LiveCell™ imaging chamber maintaining 5% CO_2_ production and a 37°C temperature (Pathology Devices Inc., Westminster, MD). Time-lapse confocal microscopy was performed using a 60x (NA 1.4) oil objective on a Nikon TE2000 inverted wide-field microscope coupled with an EM-CCD camera (Hamamatsu Inc., Bridgewater, NJ). YFP and CellTracker™ Orange were excited with 491-nm and 561-nm diode 25mW laser lines, respectively. Confocal slices of 0.4µM through the axial cell depth were acquired every 50 seconds. All experiments were performed with identical laser output and exposure settings. Time-lapse movie editing was performed using Volocity software (PerkinElmer).

**Supplementary References**

1. Berger KH, Isberg RR (1993) Two distinct defects in intracellular growth complemented by a single genetic locus in Legionella pneumophila. Mol Microbiol 7: 7-19.

2. Zuckman DM, Hung JB, Roy CR (1999) Pore-forming activity is not sufficient for Legionella pneumophila phagosome trafficking and intracellular growth. Mol Microbiol 32: 990-1001.

3. Murata T, Delprato A, Ingmundson A, Toomre DK, Lambright DG, et al. (2006) The Legionella pneumophila effector protein DrrA is a Rab1 guanine nucleotide-exchange factor. Nat Cell Biol 8: 971-977.

4. Ninio S, Celli J, Roy CR (2009) A Legionella pneumophila effector protein encoded in a region of genomic plasticity binds to Dot/Icm-modified vacuoles. PLoS Pathog 5: e1000278.

5. Nagai H, Cambronne ED, Kagan JC, Amor JC, Kahn RA, et al. (2005) A C-terminal translocation signal required for Dot/Icm-dependent delivery of the Legionella RalF protein to host cells. Proc Natl Acad Sci U S A 102: 826-831.

6. Ren T, Zamboni DS, Roy CR, Dietrich WF, Vance RE (2006) Flagellin-deficient Legionella mutants evade caspase-1- and Naip5-mediated macrophage immunity. PLoS Pathog 2: e18.

7. Finan TM, Kunkel B, De Vos GF, Signer ER (1986) Second symbiotic megaplasmid in Rhizobium meliloti carrying exopolysaccharide and thiamine synthesis genes. J Bacteriol 167: 66-72.

8. Bardill JP, Miller JL, Vogel JP (2005) IcmS-dependent translocation of SdeA into macrophages by the Legionella pneumophila type IV secretion system. Mol Microbiol 56: 90-103.

9. Merriam JJ, Mathur R, Maxfield-Boumil R, Isberg RR (1997) Analysis of the Legionella pneumophila fliI gene: intracellular growth of a defined mutant defective for flagellum biosynthesis. Infect Immun 65: 2497-2501.

10. Coers J, Monahan C, Roy CR (1999) Modulation of phagosome biogenesis by Legionella pneumophila creates an organelle permissive for intracellular growth. Nat Cell Biol 1: 451-453.

11. Ninio S, Zuckman-Cholon DM, Cambronne ED, Roy CR (2005) The Legionella IcmS-IcmW protein complex is important for Dot/Icm-mediated protein translocation. Mol Microbiol 55: 912-926.

12. Hsu F, Zhu W, Brennan L, Tao L, Luo ZQ, et al. (2012) Structural basis for substrate recognition by a unique Legionella phosphoinositide phosphatase. Proc Natl Acad Sci U S A 109: 13567-13572.

13. Ingmundson A, Delprato A, Lambright DG, Roy CR (2007) Legionella pneumophila proteins that regulate Rab1 membrane cycling. Nature 450: 365-369.

14. Derre I, Swiss R, Agaisse H (2011) The lipid transfer protein CERT interacts with the Chlamydia inclusion protein IncD and participates to ER-Chlamydia inclusion membrane contact sites. PLoS Pathog 7: e1002092.

15. Rouschop KM, Sylva M, Teske GJ, Hoedemaeker I, Pals ST, et al. (2006) Urothelial CD44 facilitates Escherichia coli infection of the murine urinary tract. J Immunol 177: 7225-7232.
